# Gas-Sensing Performance of Metal Oxide Heterojunction Materials for SF_6_ Decomposition Gases: A DFT Study

**DOI:** 10.3390/ijms25158009

**Published:** 2024-07-23

**Authors:** Tingting Zeng, Donglin Ma, Yingang Gui

**Affiliations:** 1 College of Physics and Engineering, Chengdu Normal University, Chengdu 611130, China; 073022@cdnu.edu.cn (T.Z.); 073018@cdnu.edu.cn (D.M.); 2 College of Engineering and Technology, Southwest University, Chongqing 400715, China

**Keywords:** SF_6_ decomposition gasses, metal oxide heterojunction, gas-sensing properties, DFT calculation

## Abstract

The online monitoring of GIS equipment can be realized through detecting SF_6_ decomposition gasses. Metal oxide heterojunctions are widely used as gas-sensing materials. In this study, the structural and electrical properties of In_2_O_3_-ZnO and TiO_2_-ZnO heterojunctions were analyzed based on density functional theory calculations. After heterojunction structural optimization, the electrical conductivity of these two heterojunctions was enhanced compared to each intrinsic model, and the electrical conductivity is ranked as follows: In_2_O_3_-ZnO heterojunction > TiO_2_-ZnO heterojunction. The gas-sensing response of these two heterojunctions to four SF_6_ decomposition gasses, H_2_S, SO_2_, SOF_2_, and SO_2_F_2_, was investigated. For gas adsorption systems, the adsorption energy, charge transfer, density of states, charge difference density, and frontier molecular orbitals were calculated to analyze the adsorption and gas-sensing performance. For gas adsorption on the In_2_O_3_-ZnO heterojunction surface, the induced conductivity changes are in the following order: H_2_S > SO_2_F_2_ > SOF_2_ > SO_2_. For gas adsorption on the TiO_2_-ZnO heterojunction surface, H_2_S and SOF_2_ increase conductivity, and SO_2_ and SO_2_F_2_ decrease conductivity.

## 1. Introduction

In modern power systems, the gas insulated switchgear (GIS) is an essential critical piece of equipment to ensure the safe and stable operation of the power grid [1]. Sulfur hexafluoride gas (SF_6_) is widely used in GIS equipment due to its excellent insulation and arc extinguishing performance [2]. However, during the long-term operation of GIS equipment, external environmental factors (such as overvoltage and high temperature) and inherent insulation defects of the equipment itself may lead to internal discharge and overheating faults [3]; furthermore, this can lead to SF_6_ decomposition, which has a serious impact on GIS reliability. Therefore, in order to ensure the stable operation of GIS equipment, effective online monitoring measures must be taken to quickly identify and eliminate the potential insulation faults [4]. Research has confirmed that SF_6_ decomposition gasses mainly include H_2_S, SO_2_, SOF_2_, and SO_2_F_2_, and the operating state of the GIS device can be reflected by detecting the presence and concentration of these gasses [5,6]. Therefore, it is necessary to explore gas-sensitive materials with high sensitivity and selectivity for SF_6_ decomposition gasses detection.

In recent years, the adsorption research on SF_6_ decomposition gas mainly focuses on graphene, two-dimensional metal sulfur compounds (TMDCs), metal oxides, and surface-modified metal oxides [7,8,9]. Among them, metal oxides are widely used materials with the characteristics of low cost, low power consumption, and easy synthesis for gas detection [10], including ZnO, TiO_2_, In_2_O_3_, SnO_2_, and WO_3_. ZnO is an n-type semiconductor, which is widely used for detecting toxic and hazardous gasses due to its wide bandgap and high electron mobility [11]. At the same time, researchers have also proposed various forms of nanostructures, such as nanosheets [12], nanorings [13], nanowires [14], and nanorods [15], to improve the sensitivity, selectivity, and stability of the ZnO-based gas sensor, and to reduce the working temperature and reaction recovery time. Razavi et al. prepared ZnO@TiO_2_ nanocomposites and verified their potential in the determination of hydrazine (N_2_H_4_) [16]. The research showed that TiO_2_ exhibits high selectivity and sensitivity towards small gas molecules [17,18,19]. Shooshtariet al. prepared an electronic nose based on carbon nanotube-titanium dioxide hybrid nanostructures for the detection and discrimination of volatile organic compounds (VOCs) [20]. In addition, In_2_O_3_ is also widely used in gas detection and monitoring due to its suitable bandgap and good chemical stability, and excellent gas-sensing performance [21,22]. The gas-sensing property of In_2_O_3_ can also be improved through surface metal modification or the formation of heterojunctions [23]. However, intrinsic metal oxides have limited gas sensitivity to specific gasses, and metal oxide heterojunctions are often used to improve the gas sensitivity of metal oxides-based gas sensors due to Fermi level effects and synergistic effects between different metal oxides [24,25]. Cheng et al. reported an ammonia sensor based on the ZnO/CuO heterojunction using a hydrothermal method, which effectively improved the gas-sensing property of the ZnO sensor for NH_3_ at room temperature [26].

In order to improve the sensitivity and selectivity of gas-sensitive materials towards the SF_6_ decomposition gasses of H_2_S, SO_2_, SOF_2_, and SO_2_F_2_, this study adopts In_2_O_3_-ZnO and TiO_2_-ZnO heterojunctions as gas-sensitive materials, and studies their gas-sensing properties to SF_6_ decomposition gasses based on density functional theory (DFT). Firstly, the structural models of In_2_O_3_-ZnO and TiO_2_-ZnO heterojunctions were constructed and optimized, and then the structural and energy band properties were analyzed. Secondly, the adsorption structures of different SF_6_ decomposition gasses on the heterojunction surface were constructed and optimized. Finally, by analyzing the density of states (DOS) and charge difference density (CDD) of gas adsorption systems, the adsorption performance of the metal oxide heterojunction materials for SF_6_ decomposition gasses was analyzed. The research results play an important role in promoting the application of gas-sensing technology in the online monitoring of power insulation equipment.

## 2. Results and Discussion

### 2.1. Adsorption Properties of In_2_O_3_-ZnO Heterojunction on SF_6_ Decomposition Gases

#### 2.1.1. Construction and Energy Band Analysis of In_2_O_3_-ZnO Heterojunction Model

**Figure 1:**
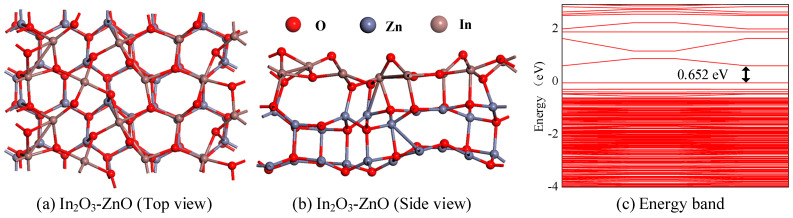
(**a**,**b**) The structure of In_2_O_3_-ZnO heterojunction, (**c**) the band structure of In_2_O_3_-ZnO heterojunction.

In the section, the most stable structure of the In_2_O_3_-ZnO heterojunction model was constructed and optimized, as shown in Figure 1a,b. The In_2_O_3_-ZnO heterojunction contains a total of 120 atoms, including 40 atoms in the In_2_O_3_ layer and 80 atoms in the ZnO heterojunction layer. The ZnO heterojunction crystal plane is placed in the lower layer, and the In_2_O_3_ crystal plane is placed in the upper layer. The lattice parameters of the ZnO heterojunction are as follows: a = 11.2558 Å, b = 16.2464 Å, and γ = 90°. The lattice parameters of In_2_O_3_ are as follows: a = 11.0324 Å, b = 16.0283 Å, and γ = 90°. The lattice parameters of the constructed In_2_O_3_-ZnO heterojunction are as follows: a = 11.1441 Å, b = 16.1373 Å, and γ = 90°. In heterostructures, there is a formation of chemical bonds between the In_2_O_3_ layer and the ZnO layer, which implies a strong interaction force between the two metal oxides. In addition, its enormous formation energy (−18.017 eV) and significant geometric deformation also prove that the formation between the two materials is highly stable. The energy band structure of the In_2_O_3_-ZnO heterojunction shown in Figure 1c intuitively displays the electron property of the heterojunction. According to the calculation results, the energy required for electrons to transition from the valence band to the conduction band is 0.652 eV. Compared to the intrinsic In_2_O_3_ (1.538 eV) and intrinsic ZnO heterojunction (1.769 eV), the significant decrease in energy indicates that the conductivity of the heterojunction is superior.

#### 2.1.2. Adsorption Properties of In_2_O_3_-ZnO Heterojunction

- Gas adsorption structure analysis

**Figure 2:**
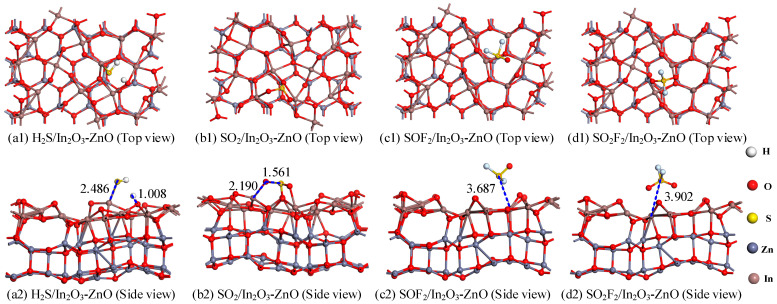
The gasses adsorption structures of (**a1**,**a2**) H_2_S, (**b1**,**b2**) SO_2_, (**c1**,**c2**) SOF_2_, and (**d1**,**d2**) SO_2_F_2_ on In_2_O_3_-ZnO heterojunction.

Figure 2 shows the most stable adsorption structures for H_2_S, SO_2_, SOF_2_, and SO_2_F_2_ on the In_2_O_3_-ZnO heterojunction surface, and Table 1 lists the corresponding adsorption parameters. For the H_2_S adsorption system shown in Figure 2(a1,a2), the strong binding force between the O and H atoms leads to the breaking of the H-S bond, resulting in the formation of a new H-O bond (1.008 Å); here, a new chemical bond is formed between the S atom and the nearest In atom with a length of 2.486 Å. As listed in Table 1, the adsorption energy of the H_2_S adsorption system reaches −1.924 eV, indicating chemical adsorption. H_2_S molecules act as electron contributors and transfer the 0.255 |*e*| charge to the In_2_O_3_-ZnO heterojunction, which is consistent with the reducibility properties of H_2_S. As shown in Figure 2(b1,b2), the O atoms of the SO_2_ molecule form chemical bonds with In atoms, while the S atom of the SO_2_ molecule forms chemical bonds with O atoms, resulting in varying degrees of elongation of the S-O bond in SO_2_. Meanwhile, the system has an adsorption energy of −1.992 eV and a charge transfer capacity of −0.361 |*e*|, indicating a strong binding force between the SO_2_ molecules and the In_2_O_3_-ZnO heterojunction. For the adsorption systems of SOF_2_ and SO_2_F_2_, shown in Figure 2(c1,c2,d1,d2), there is a significant distance between the gas molecules and heterojunctions; gas molecules can easily desorb from the surface of the In_2_O_3_-ZnO heterojunction, indicating that the adsorption process is physical adsorption. In addition, the small adsorption energy (SOF_2_: *E_ads_* = −0.465 eV, SO_2_F_2_: *E_ads_* = −0.501 eV) and the small amount of charge transfer (SOF_2_: *Q_t_* = 0.016 |*e*|, SO_2_F_2_: *Q_t_* = 0.011 |*e*|) also confirm this physical adsorption effect. In summary, the adsorption process of H_2_S and SO_2_ is chemical adsorption, and the molecular structure of the H_2_S gas molecules is destroyed, making it difficult to carry out the desorption process. The adsorption process of SOF_2_ and SO_2_F_2_ is physical adsorption and the adsorption energy is moderate, which together can have a certain adsorption capacity for gas molecules and make desorption possible.

- DOS, CDD, and molecular orbital analysis

**Figure 3:**
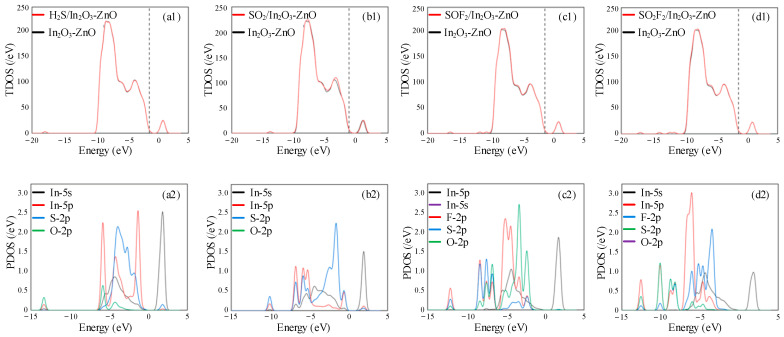
The TDOS and PDOS of (**a1**,**a2**) H_2_S, (**b1**,**b2**) SO_2_, (**c1**,**c2**) SOF_2_, and (**d1**,**d2**) SO_2_F_2_ on In_2_O_3_-ZnO heterojunction.

Figure 3 shows the density of states distribution of H_2_S, SO_2_, SOF_2_, and SO_2_F_2_ adsorption on the In_2_O_3_-ZnO heterojunction surface. In the H_2_S adsorption system shown in Figure 3(a1,a2), there is no significant change in TDOS. It can also be observed from PDOS that the hybridization phenomenon between atomic orbitals is very weak. This phenomenon indicates that the conductivity of the system remains almost unchanged before and after H_2_S adsorption. Figure 3(b1,b2) shows the TDOS and PDOS of the SO_2_ gas adsorption system. It can be observed that TDOS almost completely overlaps above the Fermi level. However, the overall density of states shows an increasing trend below the Fermi level, with the peak value increasing most significantly around −5 eV. Due to the increase in electron filling probability at this location, the conductivity of the system increases. In the PDOS distribution, there is a very obvious hybridization phenomenon between the 5p orbitals of the In atoms and the 2p orbitals of the S atoms between −10 eV and 5 eV. This phenomenon confirms the strong chemical interaction between the SO_2_ and In_2_O_3_-ZnO heterojunction, as well as the increase in TDOS near −5 eV. During the adsorption process of SOF_2_ shown in Figure 3(c1,c2), TDOS remains almost unchanged, which is consistent with its small adsorption energy and small charge transfer performance. In Figure 3(d1,d2), TDOS also keeps overlapping before and after the adsorption of SO_2_F_2_ gas, except in two new small peaks, which appear near −10 eV. In the PDOS analysis of the SO_2_F_2_ adsorption process, only In-5s orbitals and F-2p orbitals have a certain degree of hybridization, but the hybridization is not significant, which has a certain impact on improving gas adsorption performance. This result explains that compared to SOF_2_ gas molecules, the SO_2_F_2_ gas adsorption system has a greater adsorption energy.

**Figure 4:**
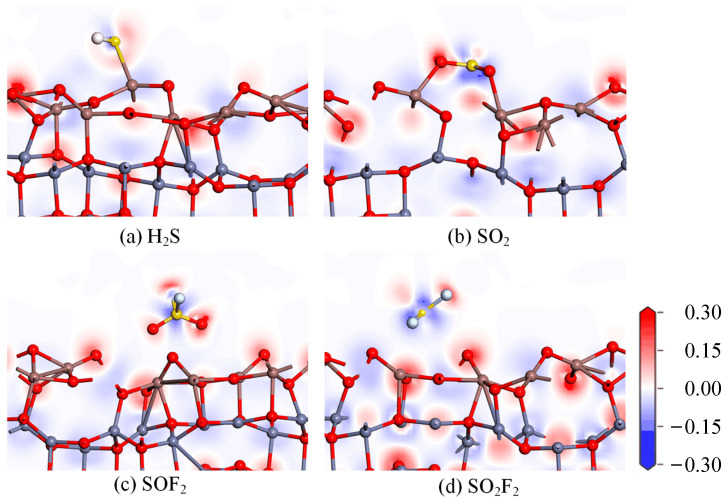
The CDD of (**a**) H_2_S, (**b**) SO_2_, (**c**) SOF_2_, and (**d**) SO_2_F_2_ on In_2_O_3_-ZnO heterojunction.

To further understand the adsorption mechanism of the In_2_O_3_-ZnO heterojunction on the four SF_6_ decomposition gasses, the charge difference density before and after adsorption were studied as shown in Figure 4; the increase in charge density is shown in red, while the decrease is shown in blue. For the SOF_2_ and SO_2_F_2_ gas molecules adsorption, there is almost no charge transfer between the In_2_O_3_-ZnO heterojunction and the gas molecules. When adsorbing H_2_S gas molecules, the S atom acts as an electron acceptor, indicating a decrease in the number of charges around the S atom. However, the red color around the S atom is very weak, and the entire H_2_S gas molecule transfers 0.255 |*e*| to the In_2_O_3_-ZnO heterojunction substrate during the adsorption process. When adsorbing SO_2_ gas molecules, the charge density around the O atom of SO_2_ molecules significantly increases, indicating that the charge transfers from the In_2_O_3_-ZnO heterojunction to SO_2_. This is attributed to the formation of chemical bonds between gas molecules and the substrate, as well as the large adsorption energy. The In_2_O_3_-ZnO heterojunction has a good adsorption effect on the SO_2_ gas molecule, but this adsorption force is not sufficient to break the original chemical bonds of the SO_2_ gas molecules. Therefore, the SO_2_ gas molecules have the potential for desorption.

**Figure 5:**
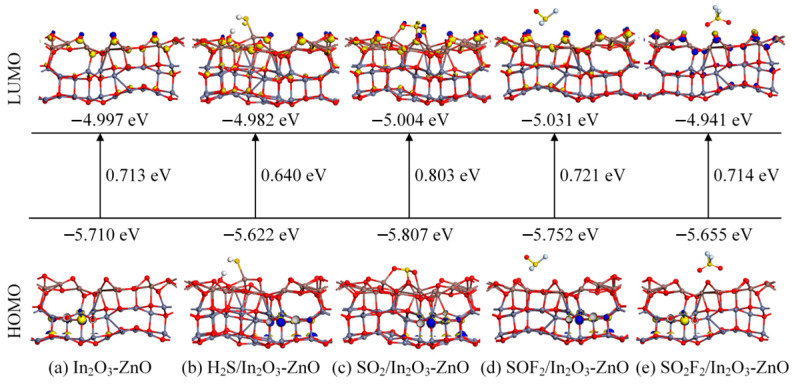
Calculated frontier molecular orbitals, HOMO and LUMO, before and after gas adsorption on In_2_O_3_-ZnO heterojunction.

Figure 5 shows the changes in the frontier molecular orbitals of theIn_2_O_3_-ZnO heterojunction before and after H_2_S, SO_2_, SOF_2_, and SO_2_F_2_ molecules adsorption, where LUMO and HOMO represent the lowest unoccupied molecular orbitals and the highest occupied molecular orbitals, respectively. It can be observed that after gas molecule adsorption, the molecular orbitals and corresponding energies change. Usually, changes in the energy gap can directly affect conductivity. During the adsorption process of H_2_S and SO_2_, there is a significant change in the energy gap value, which is related to chemical adsorption. Specifically, the conductivity of the H_2_S adsorption system increases, while the conductivity of the SO_2_ adsorption system decreases. After the adsorption of SOF_2_ and SO_2_F_2_ gas molecules, the energy gap increases, indicating a decrease in conductivity. Although this change is not very obvious, it is consistent with the previous analysis results. According to the extent of the energy gap change, the conductivity changes caused by gas adsorption are ranked in descending order: SO_2_ > H_2_S > SO_2_F_2_ > SOF_2_.

This section calculated and analyzed the adsorption performance of the In_2_O_3_-ZnO heterojunction on four gas molecules: H_2_S, SO_2_, SOF_2_, and SO_2_F_2_. Firstly, the most stable heterostructure and gas adsorption model were structured; each structure has a negative binding or adsorption energy, indicating a spontaneously performed reaction. There is a formation of chemical bonds in the adsorption process of H_2_S and SO_2_, indicating a chemical adsorption, while the adsorption of SOF_2_ and SO_2_F_2_ belongs to physical adsorption with small adsorption energy and longer adsorption distance. According to the density of states and molecular orbital theory analysis, the conductivity of the H_2_S adsorption system increases, while the conductivity of the SO_2_ adsorption system decreases. However, in the H_2_S adsorption system, the structure of gas molecules is disrupted, and cannot be desorbed from the In_2_O_3_-ZnO heterojunction surface. In the SO_2_ adsorption system, the original chemical bonds of SO_2_ gas molecules keep intact, showing a certain desorption potential. Therefore, the In_2_O_3_-ZnO heterojunction can serve as an adsorbent material for H_2_S gas molecules, while it can also be used as a gas-sensitive material for SO_2_ gas molecules.

### 2.2. Adsorption Properties of TiO_2_-ZnO Heterojunction on SF_6_ Decomposition Gases

#### 2.2.1. Construction and Energy Band Analysis of TiO_2_-ZnO Heterojunction Model

**Figure 6:**
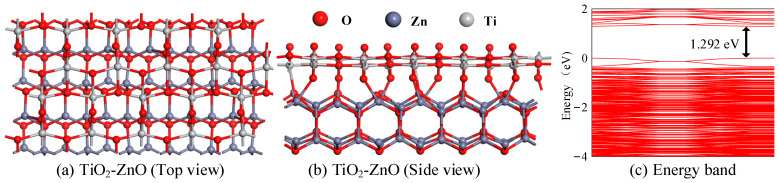
(**a**,**b**) The model of TiO_2_-ZnO heterojunction, (**c**) the band structure of TiO_2_-ZnO heterojunction.

This section constructs and optimizes the most stable model by combining the TiO_2_ and ZnO heterojunction crystal planes. Firstly, the TiO_2_ crystal plane and ZnO heterojunction crystal plane were constructed and optimized separately. The lattice parameters of the TiO_2_ crystal were as follows: a = b = 3.776 Å, c = 9.486 Å, γ = 90°. The ZnO heterojunction crystal’s lattice parameters were as follows: a = b = 3.249 Å, c = 5.205 Å, γ = 90°. Then, the TiO_2_ and ZnO heterojunction crystal planes were cut and expanded, and the obtained parameters were as follows: a = 18.880 Å, b = 10.210 Å, γ = 90° (TiO_2_ heterojunction); a = 19.496 Å, b = 10.411 Å, γ = 90° (ZnO heterojunction). In this model, the lower layer is the TiO_2_ layer, and the upper layer is the ZnO heterojunction layer. There are a total of 156 atoms, including 96 atoms in the ZnO layer and 60 atoms in the TiO_2_ layer. Figure 6a,b shows the structure of TiO_2_-ZnO heterojunction after structural optimization, with a binding energy of 12.819 eV. In the model, the TiO_2_ layer and the ZnO layer are connected by chemical bonds. In addition, the formation of chemical bonds makes it easier for electrons to migrate from the ZnO heterojunction layer to the TiO_2_ layer, as evidenced by the small energy band (1.292 eV), shown in Figure 6c. Therefore, the conductivity of the TiO_2_-ZnO heterojunction is enhanced.

#### 2.2.2. Adsorption Properties of TiO_2_-ZnO Heterojunction

- Gas adsorption structure analysis

**Figure 7:**
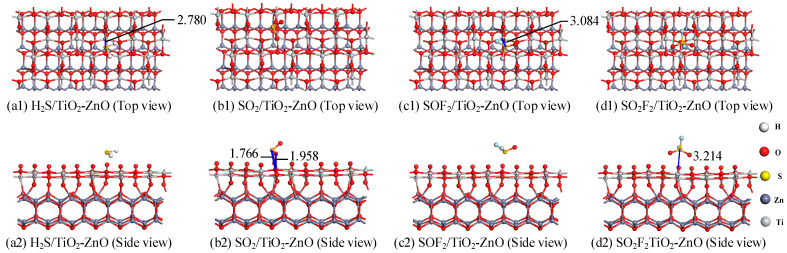
The gasses adsorption structures of (**a1**,**a2**) H_2_S, (**b1**,**b2**) SO_2_, (**c1**,**c2**) SOF_2_, and (**d1**,**d2**) SO_2_F_2_ on TiO_2_-ZnO heterojunction.

As shown in Figure 7, the most stable adsorption models are calculated by adjusting the various adsorption sites of gas molecules. As listed in Table 2, the H_2_S gas adsorption system has the maximum adsorption (−0.788 eV) and the maximum charge transfer amount (0.249 |*e*|). From Figure 7(a1,a2), it can be observed that the shortest distance between the S atoms and Ti atoms is 2.780 Å, and the long adsorption distance makes it impossible to form a chemical bond between the two. In the adsorption system of the TiO_2_-ZnO heterojunction for the four gasses, only chemical bonds form between the SO_2_ gas molecule and the substrate material, while the other three gas molecules still maintained a relatively long adsorption distance from the TiO_2_-ZnO heterojunction substrate. In the adsorption system of SOF_2_ and SO_2_F_2_, they have a long adsorption distance, small adsorption energy, and a small amount of charge transfer. Therefore, the adsorption of SOF_2_ and SO_2_F_2_ gas molecules on the TiO_2_-ZnO heterojunction is physical adsorption. In addition, only the charge transfer amount of the SO_2_ adsorption system is negative, signifying that gas molecules act as electron acceptors to obtain electrons from the substrate. It is the formation of chemical bonds (S-O: 1.766 Å; Ti-O: 1.958 Å) that facilitate electron migration, allowing SO_2_ gas molecules to form a whole with the TiO_2_ layer, and obtain more electrons from the ZnO heterojunction layer. In the other three adsorption systems, the TiO_2_ layer acts as a stronger electron acceptor, causing gas molecules to lose electrons. In the above gas adsorption, the molecular structure of the gas molecules is not disrupted. Therefore, under certain conditions, the four gas molecules can desorb from the surface of the TiO_2_-ZnO heterojunction.

- DOS, CDD, and molecular orbital analysis

**Figure 8:**
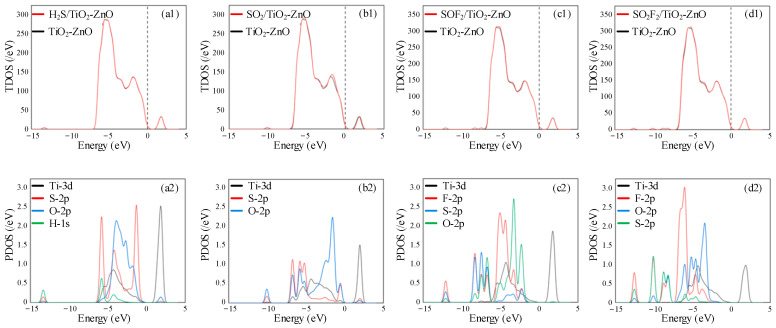
The TDOS and PDOS of (**a1**,**a2**) H_2_S, (**b1**,**b2**) SO_2_, (**c1**,**c2**) SOF_2_, and (**d1**,**d2**) SO_2_F_2_ on TiO_2_-ZnO heterojunction.

**Figure 9:**
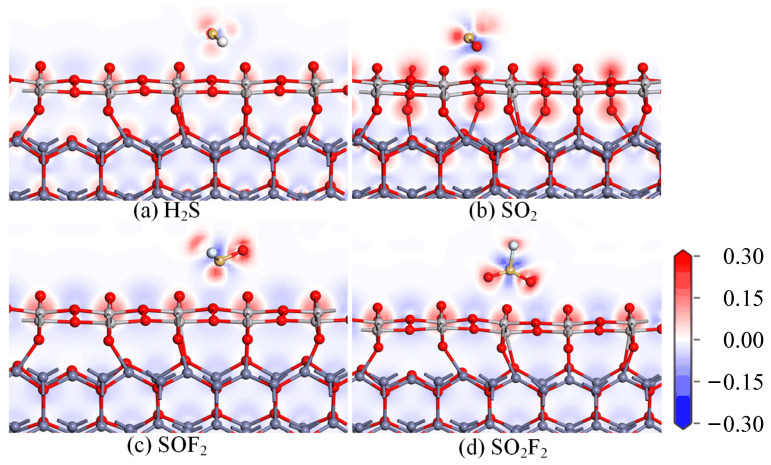
The CDD of (**a**) H_2_S, (**b**) SO_2_, (**c**) SOF_2_, and (**d**) SO_2_F_2_ on TiO_2_-ZnO heterojunction.

To investigate the gas-sensing performance of the TiO_2_-ZnO heterojunction on H_2_S, SO_2_, SOF_2_, and SO_2_F_2_ gas molecules, the density of states distribution of the four gas adsorption systems was analyzed, as shown in Figure 8. There is no significant change in TDOS before and after the four gas molecules’ adsorption on the surface of the TiO_2_-ZnO heterojunction. From PDOS analysis, it can be seen that the degree of atomic orbital hybridization is very weak. Therefore, it is not possible to accurately determine the changes in conductivity from the density of states analysis. Figure 9 shows the distribution of charges upon gas adsorption. From Figure 9a, it can be observed that the electron density around H_2_S gas is relatively low, which is consistent with the conclusion that the H_2_S molecule acts as an electron donor. In Figure 9b, the blue color is around the S atom of the SO_2_ gas molecule and the red color is around the O atoms of the TiO_2_ layer, indicating that SO_2_ receives electrons from the TiO_2_ layer. This conclusion stays consistent with the charge transfer of SO_2_ of−0.142 |*e*| during the adsorption process. In the SOF_2_ and SO_2_F_2_ adsorption systems, there is no significant change in the distribution of electrons, indicating a small amount of charge transfer.

**Figure 10:**
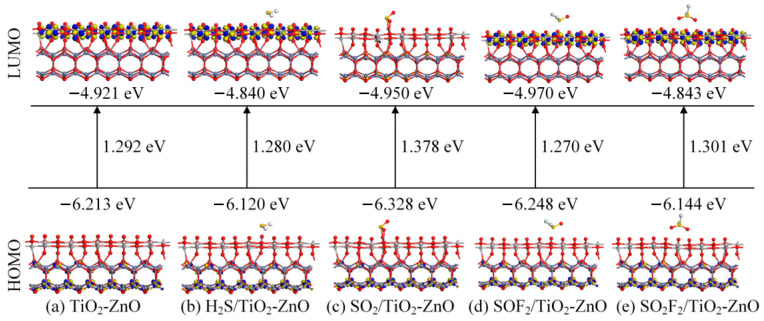
Calculated frontier molecular orbitals, HOMO and LUMO, before and after gas adsorption on TiO_2_-ZnO heterojunction.

Figure 10 shows the frontier molecular orbitals’ distribution and the related energy of the gas molecules before and after adsorption on the surface of the TiO_2_-ZnO heterojunction. Before gas adsorption, LUMO is mainly distributed on the TiO_2_ layer, while HOMO is mainly distributed on the ZnO heterojunction layer, and both are evenly distributed. The energy corresponding to LUMO is −4.921 eV, and the energy corresponding to HOMO is −6.213 eV. In the three gas adsorption systems of H_2_S, SOF_2_, and SO_2_F_2_, the energy gap only undergoes slight changes, within a range of less than 0.03 eV. The conductivity of the H_2_S and SOF_2_ gas adsorption systems increases, while the conductivity of the SO_2_F_2_ gas adsorption systems decreases. In the SO_2_ gas adsorption system, the distribution of LUMO on the TiO_2_ layer becomes thinner, followed by a significant increase in the energy gap value, which is 0.086 eV. This means there is a significant increase in conductivity after gas adsorption, providing a theoretical basis for the development of resistive chemical sensors.

This section investigated the gas-sensing response of TiO_2_-ZnO heterojunctions to four gas molecules. Based on DFT calculations, the most stable heterostructure and gas adsorption model were calculated, and the gas-sensing performance of the TiO_2_-ZnO heterojunction heterostructure on four gas molecules was explored by analyzing density of states, CDD, and the HOMO-LUMO gap. The calculation results show that the TiO_2_ and ZnO crystal planes can form stable heterojunctions with high formation energy. The adsorption type of H_2_S, SOF_2_, and SO_2_F_2_ by the TiO_2_-ZnO heterojunction is physical adsorption, while the adsorption type of SO_2_ is chemical adsorption. The conductivity of the TiO_2_-ZnO heterojunction increases after adsorbing H_2_S and SOF_2_ gas molecules; after adsorbing SO_2_ and SO_2_F_2_, there is a decrease in conductivity. Specifically, the change in conductivity is most significant after the adsorption of SO_2_, indicating that the TiO_2_-ZnO heterojunction can effectively detect SO_2_ gas.

## 3. Methods

All calculations were performed based on DFT [27]. Specifically, the generalized gradient approximation method with a GGA-PBE function was used for geometric optimization and energy calculation [28], while the Tkatchenko–Scheffler method was used to correct the van der Waals force to obtain accurate results [29]. In addition, a DFT semi-core pseudopotential (DSSP) was adopted and its value was set to 6 [30]. The K-points in the Brillouin area were set to 5 × 5 × 1. The energy convergence accuracy, maximum stress, and displacement were set to 2.721 × 10^−4^ eV, 0.109 eV/Å, and 0.005 Å, respectively [31,32]. To eliminate the influence of interlayer interactions, the thickness of the vacuum layer was set to 20 Å.

## 4. Conclusions

In order to achieve the online monitoring of GIS faults, this study analyzed the adsorption and gas-sensing properties of In_2_O_3_-ZnO and TiO_2_-ZnO heterojunctions on four SF_6_ decomposition gasses (H_2_S, SO_2_, SOF_2_, and SO_2_F_2_). Compared to intrinsic In_2_O_3_ and ZnO, the band value of the In_2_O_3_-ZnO heterojunction significantly decreases. The energy band value of the TiO_2_-ZnO heterojunction is larger than that of the In_2_O_3_-ZnO heterojunction. The In_2_O_3_-ZnO heterojunction shows large adsorption energy for H_2_S and SO_2_ via chemical adsorption, and small adsorption energy for SOF_2_ and SO_2_F_2_ via physical adsorption. After gas adsorption, the conductivity of the H_2_S adsorption system increases, while the conductivity of the SO_2_ adsorption system decreases. The In_2_O_3_-ZnO heterojunction can serve as an adsorbent material for H_2_S gas molecules, while it can also be used as a gas-sensitive material for SO_2_ gas molecules. While TiO_2_-ZnO only shows chemical adsorption for SO_2_, there is a distinct change in the conductivity of the adsorption system. The research results play an important role in exploring novel gas-sensitive materials and preparing a specific gas sensor using the online monitoring of power insulation equipment.

## Figures and Tables

**Table 1:** The adsorption parameters of H_2_S, SO_2_, SOF_2_, and SO_2_F_2_ on In_2_O_3_-ZnO heterojunction surface.

System	Distance (Å)	*E_ads_* (eV)	*Q_t_* (*e*)
H_2_S/In_2_O_3_-ZnO	In-S:2.486 H-O:1.008	−1.924	0.255
SO_2_/In_2_O_3_-ZnO	In-O:2.190 S-O:1.561	−1.992	−0.361
SOF_2_/In_2_O_3_-ZnO	In-S:3.687	−0.465	0.016
SO_2_F_2_/In_2_O_3_-ZnO	In-S:3.902	−0.501	0.011

**Table 2:** The adsorption parameters of H_2_S, SO_2_, SOF_2_, and SO_2_F_2_ on TiO_2_-ZnO heterojunction surface.

System	Distance (Å)	*E_ads_* (eV)	*Q_t_* (*e*)
H_2_S/TiO_2_-ZnO	Ti-S:2.780	−0.788	0.249
SO_2_/TiO_2_-ZnO	S-O:1.766 Ti-O:1.958	−0.608	−0.142
SOF_2_/TiO_2_-ZnO	Ti-S:3.084	−0.402	0.083
SO_2_F_2_/TiO_2_-ZnO	S-O:3.214	−0.549	0.061

## Data Availability

Data are contained within the article and Supplementary Materials.

## Supplementary Materials

The following are available online at https://www.mdpi.com/article/10.3390/ijms25158009/s1.

## Abbreviations

SF_6_	sulfur hexafluoride
GIS	gas insulated switchgear
TMDCs	two-dimensional metal sulfur compounds
VOCs	volatile organic compounds
DFT	density functional theory
DOS	density of states
TDOS	total density of states
PDOS	partial density of states
CDD	charge difference density
LUMO	lowest unoccupied molecular orbital
HOMO	highest occupied molecular orbital
GGA	generalized gradient approximation
PBE	Perdew–Burke–Ernzerhof
DSSP	DFT semi-core pseudopotential
