# Supplementary material for: Gas-Sensing Performance of Metal Oxide Heterojunction Materials for SF6 Decomposition Gases: A DFT Study
Source: Int J Mol Sci. 2024 Jul 23;25(15):8009. doi: 10.3390/ijms25158009 (PMC11311645; doi:10.3390/ijms25158009)
Supplement: Supplementary file 1 [file ijms-25-08009-s001.zip › ijms-3054736-supplementary.pdf]

In$_2$O$_3$-ZnO

==

Title In$_2$O$_3$-ZnO

Lattice type P
Space group name P 1
Space group number 1
Setting number 1

Lattice parameters

a	b	c	alpha	beta	gamma
11.14410	16.13730	27.43750	90.0000	90.0000	90.0000

Unit-cell volume = 4934.241839 Å^3

Structure parameters

			x	y	z	Occ.	U	Site	Sym.
1	Zn	Zn1	0.12208	0.05109	0.11490	1.000	0.000	1a	1
2	Zn	Zn2	0.34709	0.14429	0.08962	1.000	0.000	1a	1
3	O	O3	0.10575	0.06085	-0.02356	1.000	0.000	1a	1
4	O	O4	0.34834	0.15728	0.01272	1.000	0.000	1a	1
5	Zn	Zn5	0.01571	0.14830	-0.00129	1.000	0.000	1a	1
6	Zn	Zn6	0.26655	0.04548	0.00216	1.000	0.000	1a	1
7	O	O7	0.00362	0.14514	0.12066	1.000	0.000	1a	1
8	O	O8	0.27166	0.03723	0.07866	1.000	0.000	1a	1
9	Zn	Zn9	0.08168	0.25489	0.10897	1.000	0.000	1a	1
10	Zn	Zn10	0.33504	0.36271	0.10455	1.000	0.000	1a	1
11	O	O11	0.08618	0.24807	0.02724	1.000	0.000	1a	1
12	O	O12	0.33858	0.35968	0.02008	1.000	0.000	1a	1
13	Zn	Zn13	0.00681	0.34986	0.00958	1.000	0.000	1a	1
14	Zn	Zn14	0.25561	0.25648	0.01701	1.000	0.000	1a	1
15	O	O15	0.01574	0.37033	0.10944	1.000	0.000	1a	1
16	O	O16	0.26487	0.24797	0.11310	1.000	0.000	1a	1
17	Zn	Zn17	0.10077	0.46344	0.07346	1.000	0.000	1a	1
18	Zn	Zn18	0.36857	0.56120	0.09568	1.000	0.000	1a	1
19	O	O19	0.10297	0.44430	-0.00338	1.000	0.000	1a	1
20	O	O20	0.35880	0.54334	-0.04049	1.000	0.000	1a	1
21	Zn	Zn21	0.01908	0.55953	-0.01468	1.000	0.000	1a	1
22	Zn	Zn22	0.27197	0.45681	-0.01485	1.000	0.000	1a	1
23	O	O23	0.01787	0.56439	0.06069	1.000	0.000	1a	1
24	O	O24	0.25129	0.46799	0.10723	1.000	0.000	1a	1
25	Zn	Zn25	0.12115	0.65239	0.09266	1.000	0.000	1a	1
26	Zn	Zn26	0.36039	0.74602	0.06143	1.000	0.000	1a	1
27	O	O27	0.10872	0.65137	-0.03981	1.000	0.000	1a	1
28	O	O28	0.36056	0.75236	-0.01850	1.000	0.000	1a	1
29	Zn	Zn29	0.03688	0.74891	-0.01390	1.000	0.000	1a	1
30	Zn	Zn30	0.27501	0.64601	-0.02200	1.000	0.000	1a	1
31	O	O31	0.03279	0.74861	0.06940	1.000	0.000	1a	1
32	O	O32	0.27538	0.64254	0.05967	1.000	0.000	1a	1
33	Zn	Zn33	0.11029	0.85563	0.07259	1.000	0.000	1a	1
34	Zn	Zn34	0.36070	0.93803	0.10409	1.000	0.000	1a	1
35	O	O35	0.10324	0.86212	-0.00651	1.000	0.000	1a	1
36	O	O36	0.35006	0.96261	-0.03450	1.000	0.000	1a	1
37	Zn	Zn37	0.01769	0.95834	-0.02693	1.000	0.000	1a	1
38	Zn	Zn38	0.28109	0.85869	-0.01390	1.000	0.000	1a	1
39	O	O39	0.03971	0.94328	0.11583	1.000	0.000	1a	1
40	O	O40	0.28505	0.85385	0.06569	1.000	0.000	1a	1
41	Zn	Zn41	0.62208	0.05109	0.11490	1.000	0.000	1a	1
42	Zn	Zn42	0.84709	0.14429	0.08962	1.000	0.000	1a	1
43	O	O43	0.60575	0.06085	-0.02356	1.000	0.000	1a	1

44	O	O44	0.84834	0.15728	0.01272	1.000	0.000	1a	1
45	Zn	Zn45	0.51571	0.14830	-0.00129	1.000	0.000	1a	1
46	Zn	Zn46	0.76655	0.04548	0.00216	1.000	0.000	1a	1
47	O	O47	0.50362	0.14514	0.12066	1.000	0.000	1a	1
48	O	O48	0.77166	0.03723	0.07866	1.000	0.000	1a	1
49	Zn	Zn49	0.58168	0.25489	0.10897	1.000	0.000	1a	1
50	Zn	Zn50	0.83504	0.36271	0.10455	1.000	0.000	1a	1
51	O	O51	0.58618	0.24807	0.02724	1.000	0.000	1a	1
52	O	O52	0.83858	0.35968	0.02008	1.000	0.000	1a	1
53	Zn	Zn53	0.50681	0.34986	0.00958	1.000	0.000	1a	1
54	Zn	Zn54	0.75561	0.25648	0.01701	1.000	0.000	1a	1
55	O	O55	0.51574	0.37033	0.10944	1.000	0.000	1a	1
56	O	O56	0.76487	0.24797	0.11310	1.000	0.000	1a	1
57	Zn	Zn57	0.60077	0.46344	0.07346	1.000	0.000	1a	1
58	Zn	Zn58	0.86857	0.56120	0.09568	1.000	0.000	1a	1
59	O	O59	0.60297	0.44430	-0.00338	1.000	0.000	1a	1
60	O	O60	0.85880	0.54334	-0.04049	1.000	0.000	1a	1
61	Zn	Zn61	0.51908	0.55953	-0.01468	1.000	0.000	1a	1
62	Zn	Zn62	0.77197	0.45681	-0.01485	1.000	0.000	1a	1
63	O	O63	0.51787	0.56439	0.06069	1.000	0.000	1a	1
64	O	O64	0.75129	0.46799	0.10723	1.000	0.000	1a	1
65	Zn	Zn65	0.62115	0.65239	0.09266	1.000	0.000	1a	1
66	Zn	Zn66	0.86039	0.74602	0.06143	1.000	0.000	1a	1
67	O	O67	0.60872	0.65137	-0.03981	1.000	0.000	1a	1
68	O	O68	0.86056	0.75236	-0.01850	1.000	0.000	1a	1
69	Zn	Zn69	0.53688	0.74891	-0.01390	1.000	0.000	1a	1
70	Zn	Zn70	0.77501	0.64601	-0.02200	1.000	0.000	1a	1
71	O	O71	0.53279	0.74861	0.06940	1.000	0.000	1a	1
72	O	O72	0.77538	0.64254	0.05967	1.000	0.000	1a	1
73	Zn	Zn73	0.61029	0.85563	0.07259	1.000	0.000	1a	1
74	Zn	Zn74	0.86070	0.93803	0.10409	1.000	0.000	1a	1
75	O	O75	0.60324	0.86212	-0.00651	1.000	0.000	1a	1
76	O	O76	0.85006	0.96261	-0.03450	1.000	0.000	1a	1
77	Zn	Zn77	0.51769	0.95834	-0.02693	1.000	0.000	1a	1
78	Zn	Zn78	0.78109	0.85869	-0.01390	1.000	0.000	1a	1
79	O	O79	0.53971	0.94328	0.11583	1.000	0.000	1a	1
80	O	O80	0.78505	0.85385	0.06569	1.000	0.000	1a	1
81	In	In81	0.01063	0.14164	0.20320	1.000	0.000	1a	1
82	In	In82	0.26388	0.25169	0.19553	1.000	0.000	1a	1
83	In	In83	0.28245	0.00961	0.22320	1.000	0.000	1a	1
84	In	In84	0.00909	0.39792	0.19002	1.000	0.000	1a	1
85	O	O85	0.18062	0.09133	0.18162	1.000	0.000	1a	1
86	O	O86	-0.03383	0.01988	0.22887	1.000	0.000	1a	1
87	O	O87	0.11611	0.46654	0.23269	1.000	0.000	1a	1
88	O	O88	0.32816	0.37367	0.18771	1.000	0.000	1a	1
89	O	O89	0.35421	0.17269	0.23912	1.000	0.000	1a	1
90	O	O90	0.07845	0.26788	0.19292	1.000	0.000	1a	1
91	In	In91	0.51063	0.14164	0.20320	1.000	0.000	1a	1
92	In	In92	0.76388	0.25169	0.19553	1.000	0.000	1a	1
93	In	In93	0.78245	0.00961	0.22320	1.000	0.000	1a	1
94	In	In94	0.50909	0.39792	0.19002	1.000	0.000	1a	1
95	O	O95	0.68062	0.09133	0.18162	1.000	0.000	1a	1
96	O	O96	0.46617	0.01988	0.22887	1.000	0.000	1a	1
97	O	O97	0.61611	0.46654	0.23269	1.000	0.000	1a	1
98	O	O98	0.82816	0.37367	0.18771	1.000	0.000	1a	1
99	O	O99	0.85421	0.17269	0.23912	1.000	0.000	1a	1
100	O	O100	0.57845	0.26788	0.19292	1.000	0.000	1a	1
101	In	In101	-0.00592	0.66208	0.19439	1.000	0.000	1a	1
102	In	In102	0.25249	0.79974	0.21180	1.000	0.000	1a	1
103	In	In103	0.24028	0.51105	0.18504	1.000	0.000	1a	1
104	In	In104	0.02724	0.90913	0.19453	1.000	0.000	1a	1
105	O	O105	0.15393	0.62479	0.16284	1.000	0.000	1a	1

106 O	O106	-0.08508	0.54770	0.16855	1.000	0.000	1a	1
107 O	O107	0.17526	0.91056	0.24673	1.000	0.000	1a	1
108 O	O108	0.34006	0.90102	0.17428	1.000	0.000	1a	1
109 O	O109	0.35701	0.70681	0.23632	1.000	0.000	1a	1
110 O	O110	0.07856	0.78448	0.18557	1.000	0.000	1a	1
111 In	In111	0.49408	0.66208	0.19439	1.000	0.000	1a	1
112 In	In112	0.75249	0.79974	0.21180	1.000	0.000	1a	1
113 In	In113	0.74028	0.51105	0.18504	1.000	0.000	1a	1
114 In	In114	0.52724	0.90913	0.19453	1.000	0.000	1a	1
115 O	O115	0.65393	0.62479	0.16284	1.000	0.000	1a	1
116 O	O116	0.41492	0.54770	0.16855	1.000	0.000	1a	1
117 O	O117	0.67526	0.91056	0.24673	1.000	0.000	1a	1
118 O	O118	0.84006	0.90102	0.17428	1.000	0.000	1a	1
119 O	O119	0.85701	0.70681	0.23632	1.000	0.000	1a	1
120 O	O120	0.57856	0.78448	0.18557	1.000	0.000	1a	1

===

H_2S/In_2O_3-ZnO

===

Title H_2S/In_2O_3-ZnO

Lattice type P
Space group name P 1
Space group number 1
Setting number 1

Lattice parameters

a	b	c	alpha	beta	gamma
11.14410	16.13730	27.43750	90.0000	90.0000	90.0000

Unit-cell volume = 4934.241839 Å^3

Structure parameters

		x	y	z	Occ.	U	Site	Sym.
1 Zn	Zn1	0.12336	0.05088	0.11521	1.000	0.000	1a	1
2 Zn	Zn2	0.33426	0.16117	0.09422	1.000	0.000	1a	1
3 O	O3	0.11464	0.06217	-0.01979	1.000	0.000	1a	1
4 O	O4	0.35036	0.16368	0.01795	1.000	0.000	1a	1
5 Zn	Zn5	0.03070	0.15352	0.00114	1.000	0.000	1a	1
6 Zn	Zn6	0.27385	0.04848	0.00839	1.000	0.000	1a	1
7 O	O7	0.00985	0.14853	0.11863	1.000	0.000	1a	1
8 O	O8	0.27900	0.04562	0.08310	1.000	0.000	1a	1
9 Zn	Zn9	0.08860	0.26058	0.11041	1.000	0.000	1a	1
10 Zn	Zn10	0.37133	0.36747	0.11751	1.000	0.000	1a	1
11 O	O11	0.10230	0.25465	0.02708	1.000	0.000	1a	1
12 O	O12	0.35099	0.34412	-0.03182	1.000	0.000	1a	1
13 Zn	Zn13	0.01583	0.35480	0.00896	1.000	0.000	1a	1
14 Zn	Zn14	0.26293	0.26310	0.00093	1.000	0.000	1a	1
15 O	O15	0.00987	0.37323	0.10689	1.000	0.000	1a	1
16 O	O16	0.26673	0.26430	0.12381	1.000	0.000	1a	1
17 Zn	Zn17	0.11909	0.46140	0.07399	1.000	0.000	1a	1
18 Zn	Zn18	0.37442	0.56495	0.09034	1.000	0.000	1a	1
19 O	O19	0.11860	0.44726	-0.00187	1.000	0.000	1a	1
20 O	O20	0.36042	0.55160	-0.04117	1.000	0.000	1a	1
21 Zn	Zn21	0.02754	0.56027	-0.01013	1.000	0.000	1a	1
22 Zn	Zn22	0.28111	0.45315	-0.02921	1.000	0.000	1a	1
23 O	O23	0.02325	0.55947	0.06536	1.000	0.000	1a	1
24 O	O24	0.26934	0.46521	0.10708	1.000	0.000	1a	1
25 Zn	Zn25	0.12129	0.65155	0.09623	1.000	0.000	1a	1

26 Zn	Zn26	0.36203	0.74960	0.06370	1.000	0.000	1a	1
27 O	O27	0.10979	0.65343	-0.03782	1.000	0.000	1a	1
28 O	O28	0.36414	0.75621	-0.01585	1.000	0.000	1a	1
29 Zn	Zn29	0.03868	0.75106	-0.01242	1.000	0.000	1a	1
30 Zn	Zn30	0.27583	0.65039	-0.01959	1.000	0.000	1a	1
31 O	O31	0.03459	0.74784	0.07150	1.000	0.000	1a	1
32 O	O32	0.27204	0.64753	0.06045	1.000	0.000	1a	1
33 Zn	Zn33	0.11108	0.85519	0.07392	1.000	0.000	1a	1
34 Zn	Zn34	0.36219	0.94287	0.10640	1.000	0.000	1a	1
35 O	O35	0.10610	0.86366	-0.00452	1.000	0.000	1a	1
36 O	O36	0.35393	0.96658	-0.03053	1.000	0.000	1a	1
37 Zn	Zn37	0.02290	0.96191	-0.02425	1.000	0.000	1a	1
38 Zn	Zn38	0.28387	0.86230	-0.01136	1.000	0.000	1a	1
39 O	O39	0.03891	0.94450	0.11538	1.000	0.000	1a	1
40 O	O40	0.28643	0.85774	0.06879	1.000	0.000	1a	1
41 Zn	Zn41	0.61896	0.05861	0.11405	1.000	0.000	1a	1
42 Zn	Zn42	0.85392	0.14855	0.08668	1.000	0.000	1a	1
43 O	O43	0.60876	0.06124	-0.02591	1.000	0.000	1a	1
44 O	O44	0.86021	0.15864	0.01055	1.000	0.000	1a	1
45 Zn	Zn45	0.50948	0.14569	-0.00522	1.000	0.000	1a	1
46 Zn	Zn46	0.76893	0.04945	0.00079	1.000	0.000	1a	1
47 O	O47	0.50165	0.15698	0.11866	1.000	0.000	1a	1
48 O	O48	0.76986	0.04353	0.07794	1.000	0.000	1a	1
49 Zn	Zn49	0.58890	0.25162	0.08883	1.000	0.000	1a	1
50 Zn	Zn50	0.83234	0.36012	0.09496	1.000	0.000	1a	1
51 O	O51	0.59915	0.24576	0.01168	1.000	0.000	1a	1
52 O	O52	0.84501	0.35951	0.01462	1.000	0.000	1a	1
53 Zn	Zn53	0.50600	0.34868	-0.00157	1.000	0.000	1a	1
54 Zn	Zn54	0.76623	0.25607	0.00188	1.000	0.000	1a	1
55 O	O55	0.50989	0.35770	0.07729	1.000	0.000	1a	1
56 O	O56	0.76202	0.24895	0.11055	1.000	0.000	1a	1
57 Zn	Zn57	0.60222	0.46136	0.06651	1.000	0.000	1a	1
58 Zn	Zn58	0.86949	0.55637	0.09680	1.000	0.000	1a	1
59 O	O59	0.60698	0.44637	-0.01047	1.000	0.000	1a	1
60 O	O60	0.86923	0.54634	-0.03864	1.000	0.000	1a	1
61 Zn	Zn61	0.52394	0.56134	-0.01897	1.000	0.000	1a	1
62 Zn	Zn62	0.77404	0.45923	-0.01985	1.000	0.000	1a	1
63 O	O63	0.52537	0.56564	0.05717	1.000	0.000	1a	1
64 O	O64	0.74696	0.46417	0.10575	1.000	0.000	1a	1
65 Zn	Zn65	0.62410	0.65170	0.09256	1.000	0.000	1a	1
66 Zn	Zn66	0.86206	0.74745	0.06369	1.000	0.000	1a	1
67 O	O67	0.61610	0.65543	-0.03952	1.000	0.000	1a	1
68 O	O68	0.86311	0.75471	-0.01590	1.000	0.000	1a	1
69 Zn	Zn69	0.54016	0.74992	-0.01187	1.000	0.000	1a	1
70 Zn	Zn70	0.78141	0.64734	-0.02058	1.000	0.000	1a	1
71 O	O71	0.53436	0.74999	0.07244	1.000	0.000	1a	1
72 O	O72	0.78189	0.64186	0.06178	1.000	0.000	1a	1
73 Zn	Zn73	0.61111	0.85748	0.07527	1.000	0.000	1a	1
74 Zn	Zn74	0.85900	0.94434	0.10325	1.000	0.000	1a	1
75 O	O75	0.60646	0.86298	-0.00400	1.000	0.000	1a	1
76 O	O76	0.85535	0.96381	-0.03239	1.000	0.000	1a	1
77 Zn	Zn77	0.52190	0.95817	-0.02552	1.000	0.000	1a	1
78 Zn	Zn78	0.78395	0.86109	-0.01144	1.000	0.000	1a	1
79 O	O79	0.54090	0.94919	0.11702	1.000	0.000	1a	1
80 O	O80	0.78594	0.85526	0.06791	1.000	0.000	1a	1
81 In	In81	0.02256	0.14326	0.20199	1.000	0.000	1a	1
82 In	In82	0.23713	0.30595	0.20315	1.000	0.000	1a	1
83 In	In83	0.26977	0.00077	0.22215	1.000	0.000	1a	1
84 In	In84	-0.01600	0.40858	0.18536	1.000	0.000	1a	1
85 O	O85	0.18975	0.09146	0.18110	1.000	0.000	1a	1
86 O	O86	-0.02360	0.02041	0.22677	1.000	0.000	1a	1
87 O	O87	0.14682	0.42857	0.21895	1.000	0.000	1a	1

			x	y	z	Occ.	U	Site	Sym.
88	O	O88	0.37752	0.39226	0.19118	1.000	0.000	1a	1
89	O	O89	0.33324	0.20703	0.23810	1.000	0.000	1a	1
90	O	O90	0.05889	0.27236	0.19240	1.000	0.000	1a	1
91	In	In91	0.49310	0.15348	0.19952	1.000	0.000	1a	1
92	In	In92	0.75958	0.24520	0.19136	1.000	0.000	1a	1
93	In	In93	0.78928	0.02764	0.22239	1.000	0.000	1a	1
94	In	In94	0.52253	0.38144	0.24229	1.000	0.000	1a	1
95	O	O95	0.66840	0.09915	0.18062	1.000	0.000	1a	1
96	O	O96	0.44806	0.03590	0.22757	1.000	0.000	1a	1
97	O	O97	0.60939	0.49051	0.24085	1.000	0.000	1a	1
98	O	O98	0.80789	0.37269	0.19260	1.000	0.000	1a	1
99	O	O99	0.85422	0.16344	0.23394	1.000	0.000	1a	1
100	O	O100	0.58305	0.26652	0.21186	1.000	0.000	1a	1
101	In	In101	-0.01826	0.66292	0.19453	1.000	0.000	1a	1
102	In	In102	0.25143	0.79521	0.21197	1.000	0.000	1a	1
103	In	In103	0.25853	0.51388	0.18302	1.000	0.000	1a	1
104	In	In104	0.02209	0.90556	0.19346	1.000	0.000	1a	1
105	O	O105	0.14105	0.61644	0.16729	1.000	0.000	1a	1
106	O	O106	-0.09403	0.53890	0.17408	1.000	0.000	1a	1
107	O	O107	0.16809	0.90235	0.24734	1.000	0.000	1a	1
108	O	O108	0.34378	0.90049	0.17678	1.000	0.000	1a	1
109	O	O109	0.36549	0.71256	0.24038	1.000	0.000	1a	1
110	O	O110	0.07878	0.78162	0.18259	1.000	0.000	1a	1
111	In	In111	0.50307	0.67333	0.19554	1.000	0.000	1a	1
112	In	In112	0.75274	0.81341	0.21442	1.000	0.000	1a	1
113	In	In113	0.71877	0.50514	0.18255	1.000	0.000	1a	1
114	In	In114	0.52976	0.92325	0.19648	1.000	0.000	1a	1
115	O	O115	0.66013	0.62831	0.16297	1.000	0.000	1a	1
116	O	O116	0.40904	0.57713	0.16162	1.000	0.000	1a	1
117	O	O117	0.67748	0.92885	0.24694	1.000	0.000	1a	1
118	O	O118	0.83708	0.90961	0.17379	1.000	0.000	1a	1
119	O	O119	0.85669	0.71843	0.23762	1.000	0.000	1a	1
120	O	O120	0.58104	0.79626	0.18843	1.000	0.000	1a	1
121	S	S121	-0.56455	0.32977	0.31996	1.000	0.000	1a	1
122	H	H122	-0.46901	0.27776	0.32598	1.000	0.000	1a	1
123	H	H123	-0.63315	0.23420	0.26823	1.000	0.000	1a	1

==

SO_2/In_2O_3-ZnO

==

Title SO_2/In_2O_3-ZnO

Lattice type P
Space group name P 1
Space group number 1
Setting number 1

Lattice parameters

a	b	c	alpha	beta	gamma
11.14410	16.13730	27.43750	90.0000	90.0000	90.0000

Unit-cell volume = 4934.241839 Å^3

Structure parameters

			x	y	z	Occ.	U	Site	Sym.
1	Zn	Zn1	0.11324	0.05464	0.11820	1.000	0.000	1a	1
2	Zn	Zn2	0.35256	0.14183	0.08886	1.000	0.000	1a	1
3	O	O3	0.09882	0.05304	-0.01754	1.000	0.000	1a	1

4	O	O4	0.34746	0.15279	0.01266	1.000	0.000	1a	1
5	Zn	Zn5	0.00347	0.14251	-0.00123	1.000	0.000	1a	1
6	Zn	Zn6	0.26132	0.04327	0.00620	1.000	0.000	1a	1
7	O	O7	-0.00959	0.14807	0.12241	1.000	0.000	1a	1
8	O	O8	0.26504	0.03727	0.08298	1.000	0.000	1a	1
9	Zn	Zn9	0.06821	0.25072	0.09728	1.000	0.000	1a	1
10	Zn	Zn10	0.32189	0.35705	0.10408	1.000	0.000	1a	1
11	O	O11	0.07961	0.24734	0.02057	1.000	0.000	1a	1
12	O	O12	0.33167	0.35688	0.02005	1.000	0.000	1a	1
13	Zn	Zn13	-0.00135	0.34193	-0.01071	1.000	0.000	1a	1
14	Zn	Zn14	0.25278	0.25140	0.01580	1.000	0.000	1a	1
15	O	O15	0.01396	0.36418	0.11328	1.000	0.000	1a	1
16	O	O16	0.25000	0.24081	0.10812	1.000	0.000	1a	1
17	Zn	Zn17	0.08599	0.45208	0.07033	1.000	0.000	1a	1
18	Zn	Zn18	0.35985	0.54908	0.09027	1.000	0.000	1a	1
19	O	O19	0.09608	0.43857	-0.00596	1.000	0.000	1a	1
20	O	O20	0.35581	0.53840	-0.04130	1.000	0.000	1a	1
21	Zn	Zn21	0.01374	0.55457	-0.01902	1.000	0.000	1a	1
22	Zn	Zn22	0.26589	0.45303	-0.01571	1.000	0.000	1a	1
23	O	O23	0.01627	0.55953	0.05558	1.000	0.000	1a	1
24	O	O24	0.23734	0.46522	0.10628	1.000	0.000	1a	1
25	Zn	Zn25	0.11934	0.64345	0.08755	1.000	0.000	1a	1
26	Zn	Zn26	0.35570	0.73845	0.06213	1.000	0.000	1a	1
27	O	O27	0.10562	0.64687	-0.04496	1.000	0.000	1a	1
28	O	O28	0.35256	0.74986	-0.01831	1.000	0.000	1a	1
29	Zn	Zn29	0.02826	0.74501	-0.02668	1.000	0.000	1a	1
30	Zn	Zn30	0.27241	0.64215	-0.02494	1.000	0.000	1a	1
31	O	O31	0.00977	0.74077	0.10186	1.000	0.000	1a	1
32	O	O32	0.27076	0.63542	0.05445	1.000	0.000	1a	1
33	Zn	Zn33	0.09696	0.84310	0.08168	1.000	0.000	1a	1
34	Zn	Zn34	0.34609	0.93378	0.10481	1.000	0.000	1a	1
35	O	O35	0.08840	0.84889	0.00213	1.000	0.000	1a	1
36	O	O36	0.34026	0.95508	-0.02747	1.000	0.000	1a	1
37	Zn	Zn37	0.01214	0.95096	-0.01879	1.000	0.000	1a	1
38	Zn	Zn38	0.26833	0.85308	-0.00661	1.000	0.000	1a	1
39	O	O39	0.03754	0.94266	0.12076	1.000	0.000	1a	1
40	O	O40	0.27034	0.84262	0.07142	1.000	0.000	1a	1
41	Zn	Zn41	0.61570	0.04517	0.11273	1.000	0.000	1a	1
42	Zn	Zn42	0.83098	0.14305	0.09174	1.000	0.000	1a	1
43	O	O43	0.59853	0.05479	-0.02230	1.000	0.000	1a	1
44	O	O44	0.83956	0.15231	0.01399	1.000	0.000	1a	1
45	Zn	Zn45	0.51779	0.14900	-0.00298	1.000	0.000	1a	1
46	Zn	Zn46	0.76158	0.04212	0.00224	1.000	0.000	1a	1
47	O	O47	0.50945	0.14862	0.11927	1.000	0.000	1a	1
48	O	O48	0.76943	0.03120	0.07907	1.000	0.000	1a	1
49	Zn	Zn49	0.58809	0.25903	0.10644	1.000	0.000	1a	1
50	Zn	Zn50	0.83034	0.36036	0.12036	1.000	0.000	1a	1
51	O	O51	0.58103	0.24959	0.02342	1.000	0.000	1a	1
52	O	O52	0.83548	0.33693	-0.02555	1.000	0.000	1a	1
53	Zn	Zn53	0.50119	0.35170	0.00917	1.000	0.000	1a	1
54	Zn	Zn54	0.75618	0.26004	0.01692	1.000	0.000	1a	1
55	O	O55	0.50725	0.37067	0.10684	1.000	0.000	1a	1
56	O	O56	0.76374	0.25790	0.09448	1.000	0.000	1a	1
57	Zn	Zn57	0.61061	0.46248	0.07377	1.000	0.000	1a	1
58	Zn	Zn58	0.86621	0.55390	0.09127	1.000	0.000	1a	1
59	O	O59	0.60507	0.44353	-0.00259	1.000	0.000	1a	1
60	O	O60	0.85150	0.54322	-0.04266	1.000	0.000	1a	1
61	Zn	Zn61	0.51633	0.55452	-0.01455	1.000	0.000	1a	1
62	Zn	Zn62	0.76829	0.44657	-0.02797	1.000	0.000	1a	1
63	O	O63	0.51318	0.55863	0.06113	1.000	0.000	1a	1
64	O	O64	0.75468	0.46591	0.11001	1.000	0.000	1a	1
65	Zn	Zn65	0.61522	0.64750	0.09236	1.000	0.000	1a	1

66	Zn	Zn66	0.85941	0.74132	0.06382	1.000	0.000	1a	1
67	O	O67	0.60216	0.64865	-0.03958	1.000	0.000	1a	1
68	O	O68	0.85845	0.74772	-0.01396	1.000	0.000	1a	1
69	Zn	Zn69	0.52889	0.74565	-0.01403	1.000	0.000	1a	1
70	Zn	Zn70	0.76619	0.64087	-0.01856	1.000	0.000	1a	1
71	O	O71	0.52683	0.74353	0.06897	1.000	0.000	1a	1
72	O	O72	0.76931	0.63895	0.05926	1.000	0.000	1a	1
73	Zn	Zn73	0.60454	0.85064	0.07296	1.000	0.000	1a	1
74	Zn	Zn74	0.85694	0.93443	0.10765	1.000	0.000	1a	1
75	O	O75	0.59819	0.85730	-0.00559	1.000	0.000	1a	1
76	O	O76	0.84687	0.95645	-0.03258	1.000	0.000	1a	1
77	Zn	Zn77	0.50897	0.95388	-0.02367	1.000	0.000	1a	1
78	Zn	Zn78	0.77554	0.85493	-0.01041	1.000	0.000	1a	1
79	O	O79	0.52808	0.93985	0.11274	1.000	0.000	1a	1
80	O	O80	0.78112	0.84912	0.06870	1.000	0.000	1a	1
81	In	In81	-0.01828	0.14852	0.20511	1.000	0.000	1a	1
82	In	In82	0.25226	0.23349	0.19034	1.000	0.000	1a	1
83	In	In83	0.29499	0.02864	0.22398	1.000	0.000	1a	1
84	In	In84	0.02809	0.38650	0.19449	1.000	0.000	1a	1
85	O	O85	0.16491	0.09926	0.18491	1.000	0.000	1a	1
86	O	O86	-0.05381	0.02642	0.23083	1.000	0.000	1a	1
87	O	O87	0.11835	0.47186	0.23290	1.000	0.000	1a	1
88	O	O88	0.31677	0.35683	0.18265	1.000	0.000	1a	1
89	O	O89	0.36432	0.16293	0.23285	1.000	0.000	1a	1
90	O	O90	0.07645	0.26314	0.20920	1.000	0.000	1a	1
91	In	In91	0.53324	0.14664	0.20031	1.000	0.000	1a	1
92	In	In92	0.75432	0.29062	0.23119	1.000	0.000	1a	1
93	In	In93	0.76683	-0.00134	0.22164	1.000	0.000	1a	1
94	In	In94	0.49042	0.40084	0.18410	1.000	0.000	1a	1
95	O	O95	0.69298	0.08647	0.17578	1.000	0.000	1a	1
96	O	O96	0.48244	0.02117	0.22251	1.000	0.000	1a	1
97	O	O97	0.60763	0.47258	0.22882	1.000	0.000	1a	1
98	O	O98	0.84125	0.38418	0.19351	1.000	0.000	1a	1
99	O	O99	0.83268	0.18187	0.24637	1.000	0.000	1a	1
100	O	O100	0.58865	0.27788	0.19357	1.000	0.000	1a	1
101	In	In101	0.00188	0.68371	0.17860	1.000	0.000	1a	1
102	In	In102	0.25414	0.81208	0.22288	1.000	0.000	1a	1
103	In	In103	0.22380	0.52237	0.18145	1.000	0.000	1a	1
104	In	In104	0.03073	0.92045	0.20098	1.000	0.000	1a	1
105	O	O105	0.17508	0.64120	0.15842	1.000	0.000	1a	1
106	O	O106	-0.06387	0.56136	0.15953	1.000	0.000	1a	1
107	O	O107	0.18457	0.93189	0.25095	1.000	0.000	1a	1
108	O	O108	0.33146	0.90432	0.17601	1.000	0.000	1a	1
109	O	O109	0.35812	0.71311	0.24042	1.000	0.000	1a	1
110	O	O110	0.07286	0.79412	0.20951	1.000	0.000	1a	1
111	In	In111	0.47665	0.66084	0.19277	1.000	0.000	1a	1
112	In	In112	0.74233	0.79274	0.20795	1.000	0.000	1a	1
113	In	In113	0.77448	0.55150	0.19576	1.000	0.000	1a	1
114	In	In114	0.51814	0.90404	0.19146	1.000	0.000	1a	1
115	O	O115	0.63485	0.61787	0.16257	1.000	0.000	1a	1
116	O	O116	0.40889	0.53710	0.17069	1.000	0.000	1a	1
117	O	O117	0.66432	0.89827	0.24564	1.000	0.000	1a	1
118	O	O118	0.84301	0.89439	0.17755	1.000	0.000	1a	1
119	O	O119	0.83830	0.68719	0.21806	1.000	0.000	1a	1
120	O	O120	0.56918	0.78028	0.17913	1.000	0.000	1a	1
121	S	S121	-0.33634	0.46308	0.28495	1.000	0.000	1a	1
122	O	O122	-0.30574	0.36934	0.29145	1.000	0.000	1a	1
123	O	O123	-0.22253	0.51646	0.27503	1.000	0.000	1a	1

==

SOF$_2$/In$_2$O$_3$-ZnO

==

Title SOF$_2$/In$_2$O$_3$-ZnO

Lattice type P
Space group name P 1
Space group number 1
Setting number 1

Lattice parameters

a	b	c	alpha	beta	gamma
11.14410	16.13730	27.43750	90.0000	90.0000	90.0000

Unit-cell volume = 4934.241839 Å^3

Structure parameters

			x	y	z	Occ.	U	Site	Sym.
1	Zn	Zn1	0.12265	0.05310	0.11505	1.000	0.000	1a	1
2	Zn	Zn2	0.34374	0.14764	0.08837	1.000	0.000	1a	1
3	O	O3	0.10384	0.06348	-0.02453	1.000	0.000	1a	1
4	O	O4	0.34557	0.15969	0.01156	1.000	0.000	1a	1
5	Zn	Zn5	0.01360	0.15063	-0.00208	1.000	0.000	1a	1
6	Zn	Zn6	0.26433	0.04765	0.00139	1.000	0.000	1a	1
7	O	O7	0.00478	0.14728	0.12015	1.000	0.000	1a	1
8	O	O8	0.27066	0.03937	0.07809	1.000	0.000	1a	1
9	Zn	Zn9	0.08007	0.25766	0.10744	1.000	0.000	1a	1
10	Zn	Zn10	0.33026	0.36594	0.10194	1.000	0.000	1a	1
11	O	O11	0.08389	0.25104	0.02604	1.000	0.000	1a	1
12	O	O12	0.33553	0.36199	0.01774	1.000	0.000	1a	1
13	Zn	Zn13	0.00397	0.35247	0.00795	1.000	0.000	1a	1
14	Zn	Zn14	0.25295	0.25886	0.01461	1.000	0.000	1a	1
15	O	O15	0.01455	0.37291	0.10928	1.000	0.000	1a	1
16	O	O16	0.26214	0.25112	0.11229	1.000	0.000	1a	1
17	Zn	Zn17	0.09666	0.46588	0.07212	1.000	0.000	1a	1
18	Zn	Zn18	0.36673	0.56412	0.09317	1.000	0.000	1a	1
19	O	O19	0.09982	0.44700	-0.00505	1.000	0.000	1a	1
20	O	O20	0.35637	0.54636	-0.04159	1.000	0.000	1a	1
21	Zn	Zn21	0.01630	0.56225	-0.01610	1.000	0.000	1a	1
22	Zn	Zn22	0.26880	0.45965	-0.01663	1.000	0.000	1a	1
23	O	O23	0.01340	0.56659	0.05944	1.000	0.000	1a	1
24	O	O24	0.24868	0.47200	0.10440	1.000	0.000	1a	1
25	Zn	Zn25	0.11767	0.65459	0.09084	1.000	0.000	1a	1
26	Zn	Zn26	0.35791	0.74914	0.06044	1.000	0.000	1a	1
27	O	O27	0.10624	0.65392	-0.04122	1.000	0.000	1a	1
28	O	O28	0.35863	0.75525	-0.01946	1.000	0.000	1a	1
29	Zn	Zn29	0.03426	0.75162	-0.01543	1.000	0.000	1a	1
30	Zn	Zn30	0.27246	0.64900	-0.02311	1.000	0.000	1a	1
31	O	O31	0.03088	0.75107	0.06679	1.000	0.000	1a	1
32	O	O32	0.27248	0.64588	0.05811	1.000	0.000	1a	1
33	Zn	Zn33	0.10724	0.85840	0.07111	1.000	0.000	1a	1
34	Zn	Zn34	0.35964	0.93968	0.10326	1.000	0.000	1a	1
35	O	O35	0.10090	0.86486	-0.00818	1.000	0.000	1a	1
36	O	O36	0.34803	0.96529	-0.03560	1.000	0.000	1a	1
37	Zn	Zn37	0.01512	0.96119	-0.02810	1.000	0.000	1a	1
38	Zn	Zn38	0.27855	0.86146	-0.01522	1.000	0.000	1a	1
39	O	O39	0.03745	0.94623	0.11471	1.000	0.000	1a	1
40	O	O40	0.28215	0.85692	0.06449	1.000	0.000	1a	1
41	Zn	Zn41	0.61778	0.05475	0.11414	1.000	0.000	1a	1
42	Zn	Zn42	0.84697	0.14676	0.08952	1.000	0.000	1a	1
43	O	O43	0.60343	0.06341	-0.02377	1.000	0.000	1a	1

44	O	O44	0.84692	0.16004	0.01263	1.000	0.000	1a	1
45	Zn	Zn45	0.51345	0.15129	-0.00195	1.000	0.000	1a	1
46	Zn	Zn46	0.76421	0.04867	0.00195	1.000	0.000	1a	1
47	O	O47	0.49965	0.14852	0.11936	1.000	0.000	1a	1
48	O	O48	0.76848	0.04080	0.07850	1.000	0.000	1a	1
49	Zn	Zn49	0.58006	0.25803	0.10814	1.000	0.000	1a	1
50	Zn	Zn50	0.83377	0.36507	0.10477	1.000	0.000	1a	1
51	O	O51	0.58426	0.25090	0.02627	1.000	0.000	1a	1
52	O	O52	0.83634	0.36285	0.01947	1.000	0.000	1a	1
53	Zn	Zn53	0.50453	0.35271	0.00881	1.000	0.000	1a	1
54	Zn	Zn54	0.75401	0.25922	0.01730	1.000	0.000	1a	1
55	O	O55	0.51161	0.37359	0.10570	1.000	0.000	1a	1
56	O	O56	0.76430	0.25027	0.11227	1.000	0.000	1a	1
57	Zn	Zn57	0.60036	0.46711	0.07169	1.000	0.000	1a	1
58	Zn	Zn58	0.86488	0.56350	0.09503	1.000	0.000	1a	1
59	O	O59	0.60067	0.44713	-0.00495	1.000	0.000	1a	1
60	O	O60	0.85584	0.54615	-0.04182	1.000	0.000	1a	1
61	Zn	Zn61	0.51695	0.56228	-0.01616	1.000	0.000	1a	1
62	Zn	Zn62	0.76988	0.45904	-0.01638	1.000	0.000	1a	1
63	O	O63	0.51718	0.56800	0.05926	1.000	0.000	1a	1
64	O	O64	0.74844	0.46992	0.10721	1.000	0.000	1a	1
65	Zn	Zn65	0.61935	0.65530	0.09226	1.000	0.000	1a	1
66	Zn	Zn66	0.85842	0.74857	0.06014	1.000	0.000	1a	1
67	O	O67	0.60618	0.65475	-0.04074	1.000	0.000	1a	1
68	O	O68	0.85773	0.75522	-0.01982	1.000	0.000	1a	1
69	Zn	Zn69	0.53453	0.75209	-0.01479	1.000	0.000	1a	1
70	Zn	Zn70	0.77256	0.64876	-0.02315	1.000	0.000	1a	1
71	O	O71	0.52946	0.75181	0.07055	1.000	0.000	1a	1
72	O	O72	0.77286	0.64526	0.05884	1.000	0.000	1a	1
73	Zn	Zn73	0.60869	0.85813	0.07248	1.000	0.000	1a	1
74	Zn	Zn74	0.85776	0.94160	0.10292	1.000	0.000	1a	1
75	O	O75	0.60067	0.86479	-0.00658	1.000	0.000	1a	1
76	O	O76	0.84731	0.96578	-0.03489	1.000	0.000	1a	1
77	Zn	Zn77	0.51542	0.96105	-0.02735	1.000	0.000	1a	1
78	Zn	Zn78	0.77852	0.86161	-0.01464	1.000	0.000	1a	1
79	O	O79	0.53759	0.94589	0.11569	1.000	0.000	1a	1
80	O	O80	0.78318	0.85649	0.06485	1.000	0.000	1a	1
81	In	In81	0.01389	0.14362	0.20225	1.000	0.000	1a	1
82	In	In82	0.26303	0.25691	0.19480	1.000	0.000	1a	1
83	In	In83	0.27256	0.00198	0.22102	1.000	0.000	1a	1
84	In	In84	0.00875	0.39956	0.18995	1.000	0.000	1a	1
85	O	O85	0.18763	0.09637	0.18219	1.000	0.000	1a	1
86	O	O86	-0.03007	0.02109	0.22649	1.000	0.000	1a	1
87	O	O87	0.11488	0.47076	0.23128	1.000	0.000	1a	1
88	O	O88	0.32359	0.37970	0.18437	1.000	0.000	1a	1
89	O	O89	0.36601	0.19532	0.24246	1.000	0.000	1a	1
90	O	O90	0.07708	0.27016	0.19356	1.000	0.000	1a	1
91	In	In91	0.50340	0.14653	0.20210	1.000	0.000	1a	1
92	In	In92	0.76031	0.25435	0.19554	1.000	0.000	1a	1
93	In	In93	0.78388	0.01891	0.22274	1.000	0.000	1a	1
94	In	In94	0.50476	0.40333	0.18513	1.000	0.000	1a	1
95	O	O95	0.67081	0.09349	0.18131	1.000	0.000	1a	1
96	O	O96	0.45297	0.02487	0.22712	1.000	0.000	1a	1
97	O	O97	0.61183	0.46620	0.23118	1.000	0.000	1a	1
98	O	O98	0.82789	0.37529	0.18752	1.000	0.000	1a	1
99	O	O99	0.85031	0.16777	0.23485	1.000	0.000	1a	1
100	O	O100	0.57649	0.27316	0.18850	1.000	0.000	1a	1
101	In	In101	-0.01033	0.66471	0.19279	1.000	0.000	1a	1
102	In	In102	0.24727	0.79583	0.20764	1.000	0.000	1a	1
103	In	In103	0.23874	0.51435	0.18287	1.000	0.000	1a	1
104	In	In104	0.01985	0.90737	0.19265	1.000	0.000	1a	1
105	O	O105	0.15038	0.62709	0.16103	1.000	0.000	1a	1

106 O	O106	-0.08910	0.55097	0.16806	1.000	0.000	1a	1
107 O	O107	0.16730	0.90399	0.24485	1.000	0.000	1a	1
108 O	O108	0.33846	0.89907	0.17327	1.000	0.000	1a	1
109 O	O109	0.35065	0.70406	0.23407	1.000	0.000	1a	1
110 O	O110	0.07445	0.78500	0.17933	1.000	0.000	1a	1
111 In	In111	0.49295	0.66499	0.19334	1.000	0.000	1a	1
112 In	In112	0.74985	0.80614	0.21318	1.000	0.000	1a	1
113 In	In113	0.73745	0.51314	0.18506	1.000	0.000	1a	1
114 In	In114	0.52627	0.91523	0.19482	1.000	0.000	1a	1
115 O	O115	0.65381	0.62822	0.16272	1.000	0.000	1a	1
116 O	O116	0.41460	0.55012	0.16623	1.000	0.000	1a	1
117 O	O117	0.67481	0.91962	0.24654	1.000	0.000	1a	1
118 O	O118	0.83426	0.90567	0.17311	1.000	0.000	1a	1
119 O	O119	0.85907	0.71466	0.23639	1.000	0.000	1a	1
120 O	O120	0.57501	0.78887	0.18892	1.000	0.000	1a	1
121 S	S121	0.55536	-0.67705	0.30922	1.000	0.000	1a	1
122 F	F122	0.69616	-0.70587	0.29116	1.000	0.000	1a	1
123 F	F123	0.60714	-0.59967	0.34285	1.000	0.000	1a	1
124 O	O124	0.51667	-0.74144	0.34299	1.000	0.000	1a	1

==

SO_2F_2/In_2O_3-ZnO

==

Title SO_2F_2/In_2O_3-ZnO

Lattice type P
Space group name P 1
Space group number 1
Setting number 1

Lattice parameters

a	b	c	alpha	beta	gamma
11.14410	16.13730	27.43750	90.0000	90.0000	90.0000

Unit-cell volume = 4934.241839 Å^3

Structure parameters

		x	y	z	Occ.	U	Site	Sym.
1 Zn	Zn1	0.12582	0.04897	0.11279	1.000	0.000	1a	1
2 Zn	Zn2	0.35342	0.14103	0.08744	1.000	0.000	1a	1
3 O	O3	0.11027	0.05829	-0.02577	1.000	0.000	1a	1
4 O	O4	0.35370	0.15454	0.01058	1.000	0.000	1a	1
5 Zn	Zn5	0.02036	0.14581	-0.00343	1.000	0.000	1a	1
6 Zn	Zn6	0.27106	0.04312	-0.00007	1.000	0.000	1a	1
7 O	O7	0.00729	0.14266	0.11850	1.000	0.000	1a	1
8 O	O8	0.27542	0.03490	0.07655	1.000	0.000	1a	1
9 Zn	Zn9	0.08620	0.25224	0.10672	1.000	0.000	1a	1
10 Zn	Zn10	0.34010	0.36027	0.10247	1.000	0.000	1a	1
11 O	O11	0.09085	0.24547	0.02504	1.000	0.000	1a	1
12 O	O12	0.34352	0.35708	0.01794	1.000	0.000	1a	1
13 Zn	Zn13	0.01166	0.34731	0.00739	1.000	0.000	1a	1
14 Zn	Zn14	0.26071	0.25366	0.01571	1.000	0.000	1a	1
15 O	O15	0.02057	0.36796	0.10692	1.000	0.000	1a	1
16 O	O16	0.27000	0.24469	0.11009	1.000	0.000	1a	1
17 Zn	Zn17	0.10544	0.46120	0.07118	1.000	0.000	1a	1
18 Zn	Zn18	0.37309	0.55854	0.09284	1.000	0.000	1a	1
19 O	O19	0.10766	0.44181	-0.00575	1.000	0.000	1a	1
20 O	O20	0.36341	0.54065	-0.04279	1.000	0.000	1a	1

21	Zn	Zn21	0.02397	0.55695	-0.01693	1.000	0.000	1a	1
22	Zn	Zn22	0.27680	0.45400	-0.01714	1.000	0.000	1a	1
23	O	O23	0.02286	0.56224	0.05849	1.000	0.000	1a	1
24	O	O24	0.25588	0.46567	0.10476	1.000	0.000	1a	1
25	Zn	Zn25	0.12609	0.64992	0.09062	1.000	0.000	1a	1
26	Zn	Zn26	0.36541	0.74344	0.05884	1.000	0.000	1a	1
27	O	O27	0.11330	0.64900	-0.04204	1.000	0.000	1a	1
28	O	O28	0.36522	0.74984	-0.02117	1.000	0.000	1a	1
29	Zn	Zn29	0.04160	0.74645	-0.01605	1.000	0.000	1a	1
30	Zn	Zn30	0.27977	0.64342	-0.02447	1.000	0.000	1a	1
31	O	O31	0.03722	0.74630	0.06786	1.000	0.000	1a	1
32	O	O32	0.27978	0.64020	0.05720	1.000	0.000	1a	1
33	Zn	Zn33	0.11513	0.85310	0.07056	1.000	0.000	1a	1
34	Zn	Zn34	0.36495	0.93584	0.10144	1.000	0.000	1a	1
35	O	O35	0.10786	0.85957	-0.00856	1.000	0.000	1a	1
36	O	O36	0.35467	0.96016	-0.03663	1.000	0.000	1a	1
37	Zn	Zn37	0.02229	0.95576	-0.02906	1.000	0.000	1a	1
38	Zn	Zn38	0.28565	0.85614	-0.01627	1.000	0.000	1a	1
39	O	O39	0.04416	0.94070	0.11390	1.000	0.000	1a	1
40	O	O40	0.28974	0.85115	0.06335	1.000	0.000	1a	1
41	Zn	Zn41	0.62786	0.04809	0.11328	1.000	0.000	1a	1
42	Zn	Zn42	0.85113	0.14190	0.08742	1.000	0.000	1a	1
43	O	O43	0.61078	0.05816	-0.02612	1.000	0.000	1a	1
44	O	O44	0.85284	0.15459	0.01057	1.000	0.000	1a	1
45	Zn	Zn45	0.52079	0.14552	-0.00372	1.000	0.000	1a	1
46	Zn	Zn46	0.77121	0.04278	-0.00004	1.000	0.000	1a	1
47	O	O47	0.50968	0.14206	0.11884	1.000	0.000	1a	1
48	O	O48	0.77652	0.03440	0.07660	1.000	0.000	1a	1
49	Zn	Zn49	0.58690	0.25209	0.10661	1.000	0.000	1a	1
50	Zn	Zn50	0.83978	0.36069	0.10233	1.000	0.000	1a	1
51	O	O51	0.59114	0.24522	0.02501	1.000	0.000	1a	1
52	O	O52	0.84332	0.35716	0.01775	1.000	0.000	1a	1
53	Zn	Zn53	0.51157	0.34687	0.00724	1.000	0.000	1a	1
54	Zn	Zn54	0.76051	0.25401	0.01471	1.000	0.000	1a	1
55	O	O55	0.52125	0.36749	0.10725	1.000	0.000	1a	1
56	O	O56	0.76974	0.24578	0.11114	1.000	0.000	1a	1
57	Zn	Zn57	0.60536	0.46064	0.07114	1.000	0.000	1a	1
58	Zn	Zn58	0.87351	0.55890	0.09341	1.000	0.000	1a	1
59	O	O59	0.60759	0.44136	-0.00586	1.000	0.000	1a	1
60	O	O60	0.86356	0.54081	-0.04263	1.000	0.000	1a	1
61	Zn	Zn61	0.52401	0.55665	-0.01721	1.000	0.000	1a	1
62	Zn	Zn62	0.77658	0.45419	-0.01723	1.000	0.000	1a	1
63	O	O63	0.52269	0.56158	0.05817	1.000	0.000	1a	1
64	O	O64	0.75600	0.46598	0.10470	1.000	0.000	1a	1
65	Zn	Zn65	0.62560	0.64951	0.09027	1.000	0.000	1a	1
66	Zn	Zn66	0.86515	0.74362	0.05924	1.000	0.000	1a	1
67	O	O67	0.61357	0.64853	-0.04236	1.000	0.000	1a	1
68	O	O68	0.86547	0.74979	-0.02073	1.000	0.000	1a	1
69	Zn	Zn69	0.54159	0.74613	-0.01652	1.000	0.000	1a	1
70	Zn	Zn70	0.77970	0.64344	-0.02416	1.000	0.000	1a	1
71	O	O71	0.53773	0.74567	0.06632	1.000	0.000	1a	1
72	O	O72	0.78012	0.64020	0.05743	1.000	0.000	1a	1
73	Zn	Zn73	0.61448	0.85288	0.07003	1.000	0.000	1a	1
74	Zn	Zn74	0.86559	0.93501	0.10178	1.000	0.000	1a	1
75	O	O75	0.60786	0.85939	-0.00918	1.000	0.000	1a	1
76	O	O76	0.85475	0.96000	-0.03679	1.000	0.000	1a	1
77	Zn	Zn77	0.52239	0.95574	-0.02939	1.000	0.000	1a	1
78	Zn	Zn78	0.78564	0.85604	-0.01631	1.000	0.000	1a	1
79	O	O79	0.54412	0.94070	0.11335	1.000	0.000	1a	1
80	O	O80	0.78931	0.85135	0.06326	1.000	0.000	1a	1
81	In	In81	0.01236	0.13960	0.20113	1.000	0.000	1a	1
82	In	In82	0.26909	0.24793	0.19262	1.000	0.000	1a	1

83 In	In83	0.28844	0.00933	0.22089	1.000	0.000	1a	1
84 In	In84	0.01484	0.39450	0.18753	1.000	0.000	1a	1
85 O	O85	0.18181	0.08868	0.17977	1.000	0.000	1a	1
86 O	O86	-0.03488	0.01768	0.22631	1.000	0.000	1a	1
87 O	O87	0.12316	0.46182	0.23046	1.000	0.000	1a	1
88 O	O88	0.33315	0.36974	0.18351	1.000	0.000	1a	1
89 O	O89	0.35828	0.16725	0.23579	1.000	0.000	1a	1
90 O	O90	0.08430	0.26545	0.18972	1.000	0.000	1a	1
91 In	In91	0.51797	0.13848	0.20133	1.000	0.000	1a	1
92 In	In92	0.76930	0.25075	0.19374	1.000	0.000	1a	1
93 In	In93	0.78292	0.00089	0.22028	1.000	0.000	1a	1
94 In	In94	0.51404	0.39396	0.18788	1.000	0.000	1a	1
95 O	O95	0.68916	0.08906	0.18022	1.000	0.000	1a	1
96 O	O96	0.47303	0.01631	0.22605	1.000	0.000	1a	1
97 O	O97	0.62078	0.46335	0.23025	1.000	0.000	1a	1
98 O	O98	0.83322	0.37271	0.18482	1.000	0.000	1a	1
99 O	O99	0.86367	0.17819	0.23909	1.000	0.000	1a	1
100 O	O100	0.58353	0.26480	0.19120	1.000	0.000	1a	1
101 In	In101	-0.00103	0.65961	0.19224	1.000	0.000	1a	1
102 In	In102	0.25637	0.79878	0.21013	1.000	0.000	1a	1
103 In	In103	0.24512	0.50879	0.18261	1.000	0.000	1a	1
104 In	In104	0.03180	0.90765	0.19266	1.000	0.000	1a	1
105 O	O105	0.15970	0.62295	0.16102	1.000	0.000	1a	1
106 O	O106	-0.07988	0.54553	0.16629	1.000	0.000	1a	1
107 O	O107	0.18010	0.91050	0.24463	1.000	0.000	1a	1
108 O	O108	0.34259	0.89959	0.17167	1.000	0.000	1a	1
109 O	O109	0.36466	0.70814	0.23458	1.000	0.000	1a	1
110 O	O110	0.08228	0.78250	0.18471	1.000	0.000	1a	1
111 In	In111	0.49703	0.65941	0.19161	1.000	0.000	1a	1
112 In	In112	0.75491	0.79313	0.20779	1.000	0.000	1a	1
113 In	In113	0.74501	0.50860	0.18268	1.000	0.000	1a	1
114 In	In114	0.52915	0.90421	0.19166	1.000	0.000	1a	1
115 O	O115	0.65857	0.62224	0.16070	1.000	0.000	1a	1
116 O	O116	0.41917	0.54588	0.16562	1.000	0.000	1a	1
117 O	O117	0.67635	0.90270	0.24402	1.000	0.000	1a	1
118 O	O118	0.84427	0.89567	0.17185	1.000	0.000	1a	1
119 O	O119	0.86090	0.70275	0.23422	1.000	0.000	1a	1
120 O	O120	0.58199	0.78072	0.18015	1.000	0.000	1a	1
121 S	S121	-0.63637	0.44935	0.31209	1.000	0.000	1a	1
122 O	O122	-0.64317	0.52535	0.28441	1.000	0.000	1a	1
123 O	O123	-0.60452	0.36847	0.29366	1.000	0.000	1a	1
124 F	F124	-0.75790	0.43987	0.34263	1.000	0.000	1a	1
125 F	F125	-0.55393	0.46751	0.35884	1.000	0.000	1a	1

==

TiO_2-ZnO

==

Title TiO_2-ZnO

Lattice type P
Space group name P 1
Space group number 1
Setting number 1

Lattice parameters

a	b	c	alpha	beta	gamma
19.18780	10.31040	29.67230	90.0000	90.0000	90.0000

Unit-cell volume = 5870.186532 Å^3

Structure parameters

			x	y	z	Occ.	U	Site	Sym.
1	Zn	Zn1	0.02740	0.02562	0.01867	1.000	0.000	1a	1
2	Zn	Zn2	0.10766	1.00724	0.11185	1.000	0.000	1a	1
3	O	O3	0.02550	0.19186	-0.00692	1.000	0.000	1a	1
4	O	O4	0.10979	0.18732	0.08919	1.000	0.000	1a	1
5	Zn	Zn5	0.10968	0.25719	0.02066	1.000	0.000	1a	1
6	Zn	Zn6	0.03086	0.26492	0.12271	1.000	0.000	1a	1
7	O	O7	0.10950	0.44793	0.02814	1.000	0.000	1a	1
8	O	O8	0.03493	0.44937	0.13253	1.000	0.000	1a	1
9	Zn	Zn9	0.19335	0.02590	0.01931	1.000	0.000	1a	1
10	Zn	Zn10	0.28159	1.00828	0.11097	1.000	0.000	1a	1
11	O	O11	0.19347	0.19088	-0.00738	1.000	0.000	1a	1
12	O	O12	0.27678	0.18889	0.08850	1.000	0.000	1a	1
13	Zn	Zn13	0.27683	0.25839	0.02079	1.000	0.000	1a	1
14	Zn	Zn14	0.19329	0.26472	0.11510	1.000	0.000	1a	1
15	O	O15	0.27778	0.44825	0.03103	1.000	0.000	1a	1
16	O	O16	0.19185	0.44525	0.12490	1.000	0.000	1a	1
17	Zn	Zn17	0.35839	0.02656	0.01551	1.000	0.000	1a	1
18	Zn	Zn18	0.44921	1.01077	0.10576	1.000	0.000	1a	1
19	O	O19	0.36109	0.19566	-0.00775	1.000	0.000	1a	1
20	O	O20	0.43960	0.19851	0.09233	1.000	0.000	1a	1
21	Zn	Zn21	0.44117	0.26023	0.02518	1.000	0.000	1a	1
22	Zn	Zn22	0.35624	0.26464	0.12186	1.000	0.000	1a	1
23	O	O23	0.44172	0.44674	0.04173	1.000	0.000	1a	1
24	O	O24	0.34897	0.44854	0.13814	1.000	0.000	1a	1
25	Zn	Zn25	0.52343	0.02967	0.00851	1.000	0.000	1a	1
26	Zn	Zn26	0.61051	1.01018	0.09533	1.000	0.000	1a	1
27	O	O27	0.52474	0.20622	-0.00688	1.000	0.000	1a	1
28	O	O28	0.60845	0.20223	0.09039	1.000	0.000	1a	1
29	Zn	Zn29	0.60933	0.26133	0.02264	1.000	0.000	1a	1
30	Zn	Zn30	0.52547	0.26506	0.12030	1.000	0.000	1a	1
31	O	O31	0.60996	0.44392	0.04378	1.000	0.000	1a	1
32	O	O32	0.52002	0.44890	0.14324	1.000	0.000	1a	1
33	Zn	Zn33	0.69445	0.02844	0.00618	1.000	0.000	1a	1
34	Zn	Zn34	0.77498	1.01136	0.09829	1.000	0.000	1a	1
35	O	O35	0.69425	0.20676	-0.00695	1.000	0.000	1a	1
36	O	O36	0.78073	0.20192	0.09126	1.000	0.000	1a	1
37	Zn	Zn37	0.77883	0.26085	0.02333	1.000	0.000	1a	1
38	Zn	Zn38	0.69399	0.26582	0.11734	1.000	0.000	1a	1
39	O	O39	0.77770	0.44461	0.04296	1.000	0.000	1a	1
40	O	O40	0.69527	0.44945	0.14290	1.000	0.000	1a	1
41	Zn	Zn41	0.86395	0.02922	0.01095	1.000	0.000	1a	1
42	Zn	Zn42	0.93668	1.00978	0.10965	1.000	0.000	1a	1
43	O	O43	0.86256	0.20363	-0.00673	1.000	0.000	1a	1
44	O	O44	0.94763	0.19464	0.09254	1.000	0.000	1a	1
45	Zn	Zn45	0.94573	0.25968	0.02555	1.000	0.000	1a	1
46	Zn	Zn46	0.86341	0.26580	0.12151	1.000	0.000	1a	1
47	O	O47	0.94420	0.44824	0.03809	1.000	0.000	1a	1
48	O	O48	0.86914	0.44939	0.14204	1.000	0.000	1a	1
49	Zn	Zn49	0.02343	0.52967	0.00851	1.000	0.000	1a	1
50	Zn	Zn50	0.11051	0.51018	0.09533	1.000	0.000	1a	1
51	O	O51	0.02474	0.70622	-0.00688	1.000	0.000	1a	1
52	O	O52	0.10845	0.70223	0.09039	1.000	0.000	1a	1
53	Zn	Zn53	0.10933	0.76133	0.02264	1.000	0.000	1a	1
54	Zn	Zn54	0.02547	0.76506	0.12030	1.000	0.000	1a	1
55	O	O55	0.10996	0.94392	0.04378	1.000	0.000	1a	1
56	O	O56	0.02002	0.94890	0.14324	1.000	0.000	1a	1
57	Zn	Zn57	0.19445	0.52844	0.00618	1.000	0.000	1a	1
58	Zn	Zn58	0.27498	0.51136	0.09829	1.000	0.000	1a	1

59 O	O59	0.19425	0.70676	-0.00695	1.000	0.000	1a	1
60 O	O60	0.28073	0.70192	0.09126	1.000	0.000	1a	1
61 Zn	Zn61	0.27883	0.76085	0.02333	1.000	0.000	1a	1
62 Zn	Zn62	0.19399	0.76582	0.11734	1.000	0.000	1a	1
63 O	O63	0.27770	0.94461	0.04296	1.000	0.000	1a	1
64 O	O64	0.19527	0.94945	0.14290	1.000	0.000	1a	1
65 Zn	Zn65	0.36395	0.52922	0.01095	1.000	0.000	1a	1
66 Zn	Zn66	0.43668	0.50978	0.10965	1.000	0.000	1a	1
67 O	O67	0.36256	0.70363	-0.00673	1.000	0.000	1a	1
68 O	O68	0.44763	0.69464	0.09254	1.000	0.000	1a	1
69 Zn	Zn69	0.44573	0.75968	0.02555	1.000	0.000	1a	1
70 Zn	Zn70	0.36341	0.76580	0.12151	1.000	0.000	1a	1
71 O	O71	0.44420	0.94824	0.03809	1.000	0.000	1a	1
72 O	O72	0.36914	0.94939	0.14204	1.000	0.000	1a	1
73 Zn	Zn73	0.52740	0.52562	0.01867	1.000	0.000	1a	1
74 Zn	Zn74	0.60766	0.50724	0.11185	1.000	0.000	1a	1
75 O	O75	0.52550	0.69186	-0.00692	1.000	0.000	1a	1
76 O	O76	0.60979	0.68732	0.08919	1.000	0.000	1a	1
77 Zn	Zn77	0.60968	0.75719	0.02066	1.000	0.000	1a	1
78 Zn	Zn78	0.53086	0.76492	0.12271	1.000	0.000	1a	1
79 O	O79	0.60950	0.94793	0.02814	1.000	0.000	1a	1
80 O	O80	0.53493	0.94937	0.13253	1.000	0.000	1a	1
81 Zn	Zn81	0.69335	0.52590	0.01931	1.000	0.000	1a	1
82 Zn	Zn82	0.78159	0.50828	0.11097	1.000	0.000	1a	1
83 O	O83	0.69347	0.69088	-0.00738	1.000	0.000	1a	1
84 O	O84	0.77678	0.68889	0.08850	1.000	0.000	1a	1
85 Zn	Zn85	0.77683	0.75839	0.02079	1.000	0.000	1a	1
86 Zn	Zn86	0.69329	0.76472	0.11510	1.000	0.000	1a	1
87 O	O87	0.77778	0.94825	0.03103	1.000	0.000	1a	1
88 O	O88	0.69185	0.94525	0.12490	1.000	0.000	1a	1
89 Zn	Zn89	0.85839	0.52656	0.01551	1.000	0.000	1a	1
90 Zn	Zn90	0.94921	0.51077	0.10576	1.000	0.000	1a	1
91 O	O91	0.86109	0.69566	-0.00775	1.000	0.000	1a	1
92 O	O92	0.93960	0.69851	0.09233	1.000	0.000	1a	1
93 Zn	Zn93	0.94117	0.76023	0.02518	1.000	0.000	1a	1
94 Zn	Zn94	0.85624	0.76464	0.12186	1.000	0.000	1a	1
95 O	O95	0.94172	0.94674	0.04173	1.000	0.000	1a	1
96 O	O96	0.84897	0.94854	0.13814	1.000	0.000	1a	1
97 Ti	Ti97	0.19968	0.62981	0.23232	1.000	0.000	1a	1
98 Ti	Ti98	0.09971	0.13502	0.23331	1.000	0.000	1a	1
99 Ti	Ti99	0.09811	0.40234	0.21931	1.000	0.000	1a	1
100 Ti	Ti100	0.19908	-0.09912	0.21512	1.000	0.000	1a	1
101 O	O101	0.20003	0.43858	0.21667	1.000	0.000	1a	1
102 O	O102	0.10104	-0.05642	0.21827	1.000	0.000	1a	1
103 O	O103	0.09622	0.23758	0.18342	1.000	0.000	1a	1
104 O	O104	0.19876	0.72542	0.18263	1.000	0.000	1a	1
105 O	O105	0.19881	0.08964	0.23934	1.000	0.000	1a	1
106 O	O106	0.09851	0.58398	0.23880	1.000	0.000	1a	1
107 O	O107	0.10023	0.29077	0.26596	1.000	0.000	1a	1
108 O	O108	0.19938	-0.21483	0.26315	1.000	0.000	1a	1
109 Ti	Ti109	0.40184	0.63485	0.23393	1.000	0.000	1a	1
110 Ti	Ti110	0.29742	0.13689	0.23242	1.000	0.000	1a	1
111 Ti	Ti111	0.30350	0.40470	0.21462	1.000	0.000	1a	1
112 Ti	Ti112	0.39604	-0.09604	0.21632	1.000	0.000	1a	1
113 O	O113	0.40394	0.44065	0.21961	1.000	0.000	1a	1
114 O	O114	0.29634	-0.05743	0.21840	1.000	0.000	1a	1
115 O	O115	0.29788	0.23237	0.18127	1.000	0.000	1a	1
116 O	O116	0.40616	0.73018	0.18356	1.000	0.000	1a	1
117 O	O117	0.39651	0.08669	0.23937	1.000	0.000	1a	1
118 O	O118	0.30139	0.58515	0.23637	1.000	0.000	1a	1
119 O	O119	0.30173	0.29398	0.26269	1.000	0.000	1a	1
120 O	O120	0.39415	-0.21042	0.26446	1.000	0.000	1a	1

121 Ti	Ti121	0.59971	0.63502	0.23331	1.000	0.000	1a	1
122 Ti	Ti122	0.49750	0.13205	0.23399	1.000	0.000	1a	1
123 Ti	Ti123	0.50223	0.40180	0.21653	1.000	0.000	1a	1
124 Ti	Ti124	0.59811	-0.09766	0.21931	1.000	0.000	1a	1
125 O	O125	0.60104	0.44358	0.21827	1.000	0.000	1a	1
126 O	O126	0.49599	-0.06074	0.22015	1.000	0.000	1a	1
127 O	O127	0.49543	0.22733	0.18390	1.000	0.000	1a	1
128 O	O128	0.59622	0.73758	0.18342	1.000	0.000	1a	1
129 O	O129	0.59851	0.08398	0.23880	1.000	0.000	1a	1
130 O	O130	0.50178	0.58670	0.24123	1.000	0.000	1a	1
131 O	O131	0.50310	0.28721	0.26466	1.000	0.000	1a	1
132 O	O132	0.60023	-0.20923	0.26596	1.000	0.000	1a	1
133 Ti	Ti133	0.79742	0.63689	0.23242	1.000	0.000	1a	1
134 Ti	Ti134	0.69968	0.12981	0.23232	1.000	0.000	1a	1
135 Ti	Ti135	0.69908	0.40088	0.21512	1.000	0.000	1a	1
136 Ti	Ti136	0.80350	-0.09530	0.21462	1.000	0.000	1a	1
137 O	O137	0.79634	0.44257	0.21840	1.000	0.000	1a	1
138 O	O138	0.70003	-0.06142	0.21667	1.000	0.000	1a	1
139 O	O139	0.69876	0.22542	0.18263	1.000	0.000	1a	1
140 O	O140	0.79788	0.73237	0.18127	1.000	0.000	1a	1
141 O	O141	0.80139	0.08515	0.23637	1.000	0.000	1a	1
142 O	O142	0.69881	0.58964	0.23934	1.000	0.000	1a	1
143 O	O143	0.69938	0.28517	0.26315	1.000	0.000	1a	1
144 O	O144	0.80173	-0.20602	0.26269	1.000	0.000	1a	1
145 Ti	Ti145	0.99750	0.63205	0.23399	1.000	0.000	1a	1
146 Ti	Ti146	0.90184	0.13485	0.23393	1.000	0.000	1a	1
147 Ti	Ti147	0.89604	0.40396	0.21632	1.000	0.000	1a	1
148 Ti	Ti148	1.00223	-0.09820	0.21653	1.000	0.000	1a	1
149 O	O149	0.99599	0.43926	0.22015	1.000	0.000	1a	1
150 O	O150	0.90394	-0.05935	0.21961	1.000	0.000	1a	1
151 O	O151	0.90616	0.23018	0.18356	1.000	0.000	1a	1
152 O	O152	0.99543	0.72733	0.18390	1.000	0.000	1a	1
153 O	O153	1.00178	0.08670	0.24123	1.000	0.000	1a	1
154 O	O154	0.89651	0.58669	0.23937	1.000	0.000	1a	1
155 O	O155	0.89415	0.28958	0.26446	1.000	0.000	1a	1
156 O	O156	1.00310	-0.21279	0.26466	1.000	0.000	1a	1

==

H_2S/TiO_2-ZnO

==

Title H_2S/TiO_2-ZnO

Lattice type P
Space group name P 1
Space group number 1
Setting number 1

Lattice parameters

a	b	c	alpha	beta	gamma
19.18780	10.31040	29.67230	90.0000	90.0000	90.0000

Unit-cell volume = 5870.186532 Å^3

Structure parameters

		x	y	z	Occ.	U	Site	Sym.
1 Zn	Zn1	0.02655	0.02899	0.01814	1.000	0.000	1a	1
2 Zn	Zn2	0.10689	1.01052	0.11138	1.000	0.000	1a	1
3 O	O3	0.02436	0.19509	-0.00761	1.000	0.000	1a	1

4	O	O4	0.10887	0.19015	0.08827	1.000	0.000	1a	1
5	Zn	Zn5	0.10870	0.26051	0.01962	1.000	0.000	1a	1
6	Zn	Zn6	0.03042	0.26820	0.12205	1.000	0.000	1a	1
7	O	O7	0.10862	0.45112	0.02747	1.000	0.000	1a	1
8	O	O8	0.03472	0.45253	0.13234	1.000	0.000	1a	1
9	Zn	Zn9	0.19236	0.02932	0.01875	1.000	0.000	1a	1
10	Zn	Zn10	0.28058	1.01136	0.11028	1.000	0.000	1a	1
11	O	O11	0.19238	0.19385	-0.00838	1.000	0.000	1a	1
12	O	O12	0.27561	0.19156	0.08736	1.000	0.000	1a	1
13	Zn	Zn13	0.27589	0.26152	0.01954	1.000	0.000	1a	1
14	Zn	Zn14	0.19238	0.26780	0.11395	1.000	0.000	1a	1
15	O	O15	0.27689	0.45124	0.03015	1.000	0.000	1a	1
16	O	O16	0.19100	0.44812	0.12403	1.000	0.000	1a	1
17	Zn	Zn17	0.35716	0.02941	0.01421	1.000	0.000	1a	1
18	Zn	Zn18	0.44686	1.01292	0.10444	1.000	0.000	1a	1
19	O	O19	0.36007	0.19874	-0.00900	1.000	0.000	1a	1
20	O	O20	0.43803	0.20127	0.09120	1.000	0.000	1a	1
21	Zn	Zn21	0.44008	0.26276	0.02414	1.000	0.000	1a	1
22	Zn	Zn22	0.35494	0.26741	0.12073	1.000	0.000	1a	1
23	O	O23	0.44059	0.44915	0.04132	1.000	0.000	1a	1
24	O	O24	0.34739	0.45105	0.13751	1.000	0.000	1a	1
25	Zn	Zn25	0.52305	0.03192	0.00866	1.000	0.000	1a	1
26	Zn	Zn26	0.60908	1.01134	0.09678	1.000	0.000	1a	1
27	O	O27	0.52432	0.20851	-0.00703	1.000	0.000	1a	1
28	O	O28	0.60764	0.20379	0.09048	1.000	0.000	1a	1
29	Zn	Zn29	0.60848	0.26410	0.02289	1.000	0.000	1a	1
30	Zn	Zn30	0.52393	0.26586	0.11939	1.000	0.000	1a	1
31	O	O31	0.60912	0.44656	0.04462	1.000	0.000	1a	1
32	O	O32	0.51833	0.44991	0.14284	1.000	0.000	1a	1
33	Zn	Zn33	0.69327	0.03113	0.00678	1.000	0.000	1a	1
34	Zn	Zn34	0.77415	1.01412	0.09861	1.000	0.000	1a	1
35	O	O35	0.69325	0.20932	-0.00685	1.000	0.000	1a	1
36	O	O36	0.78016	0.20473	0.09088	1.000	0.000	1a	1
37	Zn	Zn37	0.77803	0.26370	0.02304	1.000	0.000	1a	1
38	Zn	Zn38	0.69321	0.26669	0.11705	1.000	0.000	1a	1
39	O	O39	0.77713	0.44744	0.04295	1.000	0.000	1a	1
40	O	O40	0.69502	0.45062	0.14283	1.000	0.000	1a	1
41	Zn	Zn41	0.86281	0.03205	0.01096	1.000	0.000	1a	1
42	Zn	Zn42	0.93624	1.01329	0.10931	1.000	0.000	1a	1
43	O	O43	0.86167	0.20630	-0.00704	1.000	0.000	1a	1
44	O	O44	0.94705	0.19823	0.09220	1.000	0.000	1a	1
45	Zn	Zn45	0.94480	0.26272	0.02517	1.000	0.000	1a	1
46	Zn	Zn46	0.86265	0.26834	0.12129	1.000	0.000	1a	1
47	O	O47	0.94353	0.45130	0.03788	1.000	0.000	1a	1
48	O	O48	0.86882	0.45200	0.14189	1.000	0.000	1a	1
49	Zn	Zn49	0.02245	0.53306	0.00809	1.000	0.000	1a	1
50	Zn	Zn50	0.10968	0.51374	0.09467	1.000	0.000	1a	1
51	O	O51	0.02368	0.70961	-0.00720	1.000	0.000	1a	1
52	O	O52	0.10766	0.70570	0.08991	1.000	0.000	1a	1
53	Zn	Zn53	0.10850	0.76472	0.02213	1.000	0.000	1a	1
54	Zn	Zn54	0.02479	0.76840	0.12002	1.000	0.000	1a	1
55	O	O55	0.10906	0.94720	0.04326	1.000	0.000	1a	1
56	O	O56	0.01949	0.95209	0.14304	1.000	0.000	1a	1
57	Zn	Zn57	0.19353	0.53181	0.00551	1.000	0.000	1a	1
58	Zn	Zn58	0.27393	0.51480	0.09740	1.000	0.000	1a	1
59	O	O59	0.19350	0.71018	-0.00742	1.000	0.000	1a	1
60	O	O60	0.28017	0.70528	0.09081	1.000	0.000	1a	1
61	Zn	Zn61	0.27822	0.76406	0.02284	1.000	0.000	1a	1
62	Zn	Zn62	0.19332	0.76921	0.11678	1.000	0.000	1a	1
63	O	O63	0.27669	0.94788	0.04218	1.000	0.000	1a	1
64	O	O64	0.19440	0.95262	0.14249	1.000	0.000	1a	1
65	Zn	Zn65	0.36321	0.53237	0.01036	1.000	0.000	1a	1

66 Zn	Zn66	0.43523	0.51242	0.10910	1.000	0.000	1a	1
67 O	O67	0.36243	0.70710	-0.00669	1.000	0.000	1a	1
68 O	O68	0.44761	0.69744	0.09333	1.000	0.000	1a	1
69 Zn	Zn69	0.44523	0.76205	0.02647	1.000	0.000	1a	1
70 Zn	Zn70	0.36271	0.76871	0.12154	1.000	0.000	1a	1
71 O	O71	0.44263	0.95130	0.03705	1.000	0.000	1a	1
72 O	O72	0.36753	0.95239	0.14163	1.000	0.000	1a	1
73 Zn	Zn73	0.52673	0.52813	0.01918	1.000	0.000	1a	1
74 Zn	Zn74	0.60670	0.50982	0.11289	1.000	0.000	1a	1
75 O	O75	0.52545	0.69504	-0.00571	1.000	0.000	1a	1
76 O	O76	0.60895	0.69084	0.09197	1.000	0.000	1a	1
77 Zn	Zn77	0.60872	0.75997	0.02318	1.000	0.000	1a	1
78 Zn	Zn78	0.52985	0.76653	0.12638	1.000	0.000	1a	1
79 O	O79	0.60872	0.95083	0.02920	1.000	0.000	1a	1
80 O	O80	0.53144	0.95251	0.13192	1.000	0.000	1a	1
81 Zn	Zn81	0.69236	0.52841	0.01988	1.000	0.000	1a	1
82 Zn	Zn82	0.78140	0.51062	0.11079	1.000	0.000	1a	1
83 O	O83	0.69196	0.69435	-0.00589	1.000	0.000	1a	1
84 O	O84	0.77581	0.69199	0.08958	1.000	0.000	1a	1
85 Zn	Zn85	0.77576	0.76124	0.02176	1.000	0.000	1a	1
86 Zn	Zn86	0.69294	0.76813	0.11717	1.000	0.000	1a	1
87 O	O87	0.77682	0.95125	0.03139	1.000	0.000	1a	1
88 O	O88	0.69130	0.94911	0.12618	1.000	0.000	1a	1
89 Zn	Zn89	0.85770	0.52973	0.01544	1.000	0.000	1a	1
90 Zn	Zn90	0.94877	0.51386	0.10561	1.000	0.000	1a	1
91 O	O91	0.85973	0.69926	-0.00740	1.000	0.000	1a	1
92 O	O92	0.93882	0.70152	0.09223	1.000	0.000	1a	1
93 Zn	Zn93	0.94029	0.76355	0.02514	1.000	0.000	1a	1
94 Zn	Zn94	0.85589	0.76856	0.12225	1.000	0.000	1a	1
95 O	O95	0.94109	0.95013	0.04141	1.000	0.000	1a	1
96 O	O96	0.84901	0.95258	0.13777	1.000	0.000	1a	1
97 Ti	Ti97	0.19894	0.63238	0.23162	1.000	0.000	1a	1
98 Ti	Ti98	0.09918	0.13751	0.23258	1.000	0.000	1a	1
99 Ti	Ti99	0.09757	0.40469	0.21817	1.000	0.000	1a	1
100 Ti	Ti100	0.19861	-0.09639	0.21476	1.000	0.000	1a	1
101 O	O101	0.19914	0.44109	0.21548	1.000	0.000	1a	1
102 O	O102	0.10035	-0.05433	0.21814	1.000	0.000	1a	1
103 O	O103	0.09546	0.23941	0.18255	1.000	0.000	1a	1
104 O	O104	0.19827	0.72845	0.18206	1.000	0.000	1a	1
105 O	O105	0.19824	0.09222	0.23857	1.000	0.000	1a	1
106 O	O106	0.09786	0.58636	0.23802	1.000	0.000	1a	1
107 O	O107	0.09985	0.29351	0.26500	1.000	0.000	1a	1
108 O	O108	0.19869	-0.21289	0.26269	1.000	0.000	1a	1
109 Ti	Ti109	0.40073	0.63701	0.23369	1.000	0.000	1a	1
110 Ti	Ti110	0.29712	0.13943	0.23154	1.000	0.000	1a	1
111 Ti	Ti111	0.30256	0.40693	0.21323	1.000	0.000	1a	1
112 Ti	Ti112	0.39590	-0.09358	0.21682	1.000	0.000	1a	1
113 O	O113	0.40300	0.44250	0.21819	1.000	0.000	1a	1
114 O	O114	0.29592	-0.05531	0.21850	1.000	0.000	1a	1
115 O	O115	0.29695	0.23415	0.18024	1.000	0.000	1a	1
116 O	O116	0.40649	0.73417	0.18365	1.000	0.000	1a	1
117 O	O117	0.39611	0.09006	0.23878	1.000	0.000	1a	1
118 O	O118	0.30053	0.58706	0.23558	1.000	0.000	1a	1
119 O	O119	0.30092	0.29687	0.26155	1.000	0.000	1a	1
120 O	O120	0.39280	-0.20898	0.26465	1.000	0.000	1a	1
121 Ti	Ti121	0.59870	0.63260	0.23893	1.000	0.000	1a	1
122 Ti	Ti122	0.49773	0.13507	0.23329	1.000	0.000	1a	1
123 Ti	Ti123	0.50107	0.40488	0.21472	1.000	0.000	1a	1
124 Ti	Ti124	0.59800	-0.09986	0.22344	1.000	0.000	1a	1
125 O	O125	0.60033	0.44876	0.21655	1.000	0.000	1a	1
126 O	O126	0.49581	-0.05882	0.22212	1.000	0.000	1a	1
127 O	O127	0.49426	0.22887	0.18298	1.000	0.000	1a	1

128 O	O128	0.59324	0.74192	0.18808	1.000	0.000	1a	1
129 O	O129	0.59820	0.08800	0.23877	1.000	0.000	1a	1
130 O	O130	0.49953	0.58764	0.24125	1.000	0.000	1a	1
131 O	O131	0.50392	0.29138	0.26356	1.000	0.000	1a	1
132 O	O132	0.60134	-0.20615	0.27082	1.000	0.000	1a	1
133 Ti	Ti133	0.79759	0.63857	0.23248	1.000	0.000	1a	1
134 Ti	Ti134	0.69853	0.13371	0.23247	1.000	0.000	1a	1
135 Ti	Ti135	0.69886	0.40502	0.21349	1.000	0.000	1a	1
136 Ti	Ti136	0.80220	-0.09296	0.21671	1.000	0.000	1a	1
137 O	O137	0.79602	0.44400	0.21816	1.000	0.000	1a	1
138 O	O138	0.69991	-0.05920	0.21905	1.000	0.000	1a	1
139 O	O139	0.70046	0.22724	0.18237	1.000	0.000	1a	1
140 O	O140	0.79794	0.73738	0.18209	1.000	0.000	1a	1
141 O	O141	0.80073	0.08821	0.23795	1.000	0.000	1a	1
142 O	O142	0.69940	0.59185	0.23855	1.000	0.000	1a	1
143 O	O143	0.69595	0.29071	0.26282	1.000	0.000	1a	1
144 O	O144	0.80127	-0.20636	0.26409	1.000	0.000	1a	1
145 Ti	Ti145	0.99707	0.63459	0.23356	1.000	0.000	1a	1
146 Ti	Ti146	0.90106	0.13701	0.23400	1.000	0.000	1a	1
147 Ti	Ti147	0.89594	0.40562	0.21613	1.000	0.000	1a	1
148 Ti	Ti148	1.00149	-0.09550	0.21639	1.000	0.000	1a	1
149 O	O149	0.99545	0.44150	0.21948	1.000	0.000	1a	1
150 O	O150	0.90299	-0.05693	0.22018	1.000	0.000	1a	1
151 O	O151	0.90456	0.23200	0.18351	1.000	0.000	1a	1
152 O	O152	0.99418	0.73038	0.18359	1.000	0.000	1a	1
153 O	O153	1.00118	0.08932	0.24085	1.000	0.000	1a	1
154 O	O154	0.89629	0.58830	0.23953	1.000	0.000	1a	1
155 O	O155	0.89438	0.29221	0.26449	1.000	0.000	1a	1
156 O	O156	1.00275	-0.21075	0.26442	1.000	0.000	1a	1
157 S	S157	0.60136	0.52889	0.32540	1.000	0.000	1a	1
158 H	H158	0.66572	0.57931	0.33378	1.000	0.000	1a	1
159 H	H159	0.62925	0.41355	0.31123	1.000	0.000	1a	1

===

SO_2/TiO_2-ZnO

===

Title SO_2/TiO_2-ZnO

Lattice type P
Space group name P 1
Space group number 1
Setting number 1

Lattice parameters

a	b	c	alpha	beta	gamma
19.18780	10.31040	29.67230	90.0000	90.0000	90.0000

Unit-cell volume = 5870.186532 Å^3

Structure parameters

		x	y	z	Occ.	U	Site	Sym.
1 Zn	Zn1	0.03186	0.02243	0.01681	1.000	0.000	1a	1
2 Zn	Zn2	0.10943	1.00490	0.11106	1.000	0.000	1a	1
3 O	O3	0.02946	0.19036	-0.00747	1.000	0.000	1a	1
4 O	O4	0.11381	0.18520	0.08852	1.000	0.000	1a	1
5 Zn	Zn5	0.11391	0.25396	0.02033	1.000	0.000	1a	1
6 Zn	Zn6	0.03469	0.26144	0.12249	1.000	0.000	1a	1
7 O	O7	0.11328	0.44414	0.02965	1.000	0.000	1a	1

8	O	O8	0.04202	0.44626	0.13673	1.000	0.000	1a	1
9	Zn	Zn9	0.19757	0.02197	0.01895	1.000	0.000	1a	1
10	Zn	Zn10	0.28366	1.00402	0.11067	1.000	0.000	1a	1
11	O	O11	0.19718	0.18653	-0.00820	1.000	0.000	1a	1
12	O	O12	0.28232	0.18303	0.08641	1.000	0.000	1a	1
13	Zn	Zn13	0.28167	0.25321	0.01849	1.000	0.000	1a	1
14	Zn	Zn14	0.19878	0.25823	0.11369	1.000	0.000	1a	1
15	O	O15	0.28249	0.44397	0.02750	1.000	0.000	1a	1
16	O	O16	0.19961	0.43945	0.12261	1.000	0.000	1a	1
17	Zn	Zn17	0.36348	0.02156	0.01828	1.000	0.000	1a	1
18	Zn	Zn18	0.45608	1.00536	0.10836	1.000	0.000	1a	1
19	O	O19	0.36659	0.18723	-0.00792	1.000	0.000	1a	1
20	O	O20	0.44400	0.19133	0.09236	1.000	0.000	1a	1
21	Zn	Zn21	0.44519	0.25637	0.02543	1.000	0.000	1a	1
22	Zn	Zn22	0.36022	0.25816	0.12157	1.000	0.000	1a	1
23	O	O23	0.44549	0.44395	0.04070	1.000	0.000	1a	1
24	O	O24	0.35181	0.44329	0.13574	1.000	0.000	1a	1
25	Zn	Zn25	0.52689	0.02634	0.00954	1.000	0.000	1a	1
26	Zn	Zn26	0.61606	1.00701	0.09635	1.000	0.000	1a	1
27	O	O27	0.52822	0.20167	-0.00712	1.000	0.000	1a	1
28	O	O28	0.61106	0.19807	0.09031	1.000	0.000	1a	1
29	Zn	Zn29	0.61281	0.25719	0.02234	1.000	0.000	1a	1
30	Zn	Zn30	0.52904	0.26144	0.12096	1.000	0.000	1a	1
31	O	O31	0.61383	0.44029	0.04261	1.000	0.000	1a	1
32	O	O32	0.52266	0.44611	0.14171	1.000	0.000	1a	1
33	Zn	Zn33	0.69718	0.02435	0.00516	1.000	0.000	1a	1
34	Zn	Zn34	0.78071	1.00742	0.09434	1.000	0.000	1a	1
35	O	O35	0.69735	0.20290	-0.00767	1.000	0.000	1a	1
36	O	O36	0.78370	0.19924	0.08984	1.000	0.000	1a	1
37	Zn	Zn37	0.78226	0.25790	0.02194	1.000	0.000	1a	1
38	Zn	Zn38	0.69771	0.26301	0.11635	1.000	0.000	1a	1
39	O	O39	0.78107	0.44077	0.04253	1.000	0.000	1a	1
40	O	O40	0.69704	0.44706	0.14165	1.000	0.000	1a	1
41	Zn	Zn41	0.86854	0.02672	0.00843	1.000	0.000	1a	1
42	Zn	Zn42	0.94069	1.00714	0.10762	1.000	0.000	1a	1
43	O	O43	0.86704	0.20284	-0.00728	1.000	0.000	1a	1
44	O	O44	0.95151	0.19387	0.09256	1.000	0.000	1a	1
45	Zn	Zn45	0.94997	0.25694	0.02566	1.000	0.000	1a	1
46	Zn	Zn46	0.86594	0.26262	0.12059	1.000	0.000	1a	1
47	O	O47	0.94923	0.44422	0.04095	1.000	0.000	1a	1
48	O	O48	0.87150	0.44680	0.14232	1.000	0.000	1a	1
49	Zn	Zn49	0.02709	0.52623	0.01014	1.000	0.000	1a	1
50	Zn	Zn50	0.11597	0.50710	0.09718	1.000	0.000	1a	1
51	O	O51	0.02843	0.70134	-0.00681	1.000	0.000	1a	1
52	O	O52	0.11076	0.69821	0.09063	1.000	0.000	1a	1
53	Zn	Zn53	0.11241	0.75745	0.02308	1.000	0.000	1a	1
54	Zn	Zn54	0.02884	0.76191	0.12188	1.000	0.000	1a	1
55	O	O55	0.11358	0.94098	0.04303	1.000	0.000	1a	1
56	O	O56	0.02227	0.94646	0.14261	1.000	0.000	1a	1
57	Zn	Zn57	0.19690	0.52410	0.00572	1.000	0.000	1a	1
58	Zn	Zn58	0.28048	0.50644	0.09398	1.000	0.000	1a	1
59	O	O59	0.19796	0.70310	-0.00600	1.000	0.000	1a	1
60	O	O60	0.28437	0.69897	0.09265	1.000	0.000	1a	1
61	Zn	Zn61	0.28246	0.75652	0.02421	1.000	0.000	1a	1
62	Zn	Zn62	0.19689	0.76273	0.11724	1.000	0.000	1a	1
63	O	O63	0.28120	0.93979	0.04346	1.000	0.000	1a	1
64	O	O64	0.19687	0.94756	0.14193	1.000	0.000	1a	1
65	Zn	Zn65	0.36881	0.52701	0.00885	1.000	0.000	1a	1
66	Zn	Zn66	0.43971	0.50591	0.10857	1.000	0.000	1a	1
67	O	O67	0.36687	0.70350	-0.00576	1.000	0.000	1a	1
68	O	O68	0.45021	0.69357	0.09479	1.000	0.000	1a	1
69	Zn	Zn69	0.44931	0.75620	0.02787	1.000	0.000	1a	1

70	Zn	Zn70	0.36560	0.75308	0.12648	1.000	0.000	1a	1
71	O	O71	0.44976	0.94417	0.04152	1.000	0.000	1a	1
72	O	O72	0.37266	0.94748	0.14294	1.000	0.000	1a	1
73	Zn	Zn73	0.53169	0.52216	0.01758	1.000	0.000	1a	1
74	Zn	Zn74	0.61053	0.50503	0.11048	1.000	0.000	1a	1
75	O	O75	0.52777	0.69012	-0.00646	1.000	0.000	1a	1
76	O	O76	0.61341	0.68492	0.08740	1.000	0.000	1a	1
77	Zn	Zn77	0.61348	0.75436	0.01946	1.000	0.000	1a	1
78	Zn	Zn78	0.53530	0.75899	0.12265	1.000	0.000	1a	1
79	O	O79	0.61299	0.94459	0.02906	1.000	0.000	1a	1
80	O	O80	0.54318	0.94479	0.13723	1.000	0.000	1a	1
81	Zn	Zn81	0.69754	0.52283	0.01838	1.000	0.000	1a	1
82	Zn	Zn82	0.78433	0.50508	0.11071	1.000	0.000	1a	1
83	O	O83	0.69714	0.68771	-0.00850	1.000	0.000	1a	1
84	O	O84	0.78135	0.68457	0.08747	1.000	0.000	1a	1
85	Zn	Zn85	0.78132	0.75383	0.01927	1.000	0.000	1a	1
86	Zn	Zn86	0.69746	0.75997	0.11375	1.000	0.000	1a	1
87	O	O87	0.78211	0.94452	0.02738	1.000	0.000	1a	1
88	O	O88	0.69902	0.94082	0.12257	1.000	0.000	1a	1
89	Zn	Zn89	0.86360	0.52231	0.01748	1.000	0.000	1a	1
90	Zn	Zn90	0.95477	0.50749	0.10889	1.000	0.000	1a	1
91	O	O91	0.86621	0.68925	-0.00759	1.000	0.000	1a	1
92	O	O92	0.94376	0.69340	0.09290	1.000	0.000	1a	1
93	Zn	Zn93	0.94532	0.75635	0.02586	1.000	0.000	1a	1
94	Zn	Zn94	0.85966	0.76111	0.12208	1.000	0.000	1a	1
95	O	O95	0.94599	0.94446	0.03967	1.000	0.000	1a	1
96	O	O96	0.85348	0.94607	0.13439	1.000	0.000	1a	1
97	Ti	Ti97	0.19188	0.62942	0.23116	1.000	0.000	1a	1
98	Ti	Ti98	0.09528	0.13662	0.23334	1.000	0.000	1a	1
99	Ti	Ti99	0.09028	0.40375	0.21547	1.000	0.000	1a	1
100	Ti	Ti100	0.19419	-0.09880	0.21496	1.000	0.000	1a	1
101	O	O101	0.19123	0.44083	0.21229	1.000	0.000	1a	1
102	O	O102	0.09690	-0.05671	0.21939	1.000	0.000	1a	1
103	O	O103	0.09271	0.23120	0.18209	1.000	0.000	1a	1
104	O	O104	0.18401	0.72728	0.18228	1.000	0.000	1a	1
105	O	O105	0.19416	0.09091	0.23974	1.000	0.000	1a	1
106	O	O106	0.09166	0.58267	0.23951	1.000	0.000	1a	1
107	O	O107	0.09359	0.29460	0.26354	1.000	0.000	1a	1
108	O	O108	0.19921	-0.21540	0.26249	1.000	0.000	1a	1
109	Ti	Ti109	0.39669	0.62030	0.24002	1.000	0.000	1a	1
110	Ti	Ti110	0.29153	0.14258	0.23096	1.000	0.000	1a	1
111	Ti	Ti111	0.29567	0.40896	0.20912	1.000	0.000	1a	1
112	Ti	Ti112	0.38928	-0.08959	0.21114	1.000	0.000	1a	1
113	O	O113	0.39880	0.44336	0.21812	1.000	0.000	1a	1
114	O	O114	0.29077	-0.05152	0.21474	1.000	0.000	1a	1
115	O	O115	0.29214	0.23145	0.17808	1.000	0.000	1a	1
116	O	O116	0.40040	0.73259	0.18925	1.000	0.000	1a	1
117	O	O117	0.38980	0.09321	0.23828	1.000	0.000	1a	1
118	O	O118	0.29351	0.58648	0.23018	1.000	0.000	1a	1
119	O	O119	0.29328	0.30386	0.25853	1.000	0.000	1a	1
120	O	O120	0.38348	-0.19242	0.26634	1.000	0.000	1a	1
121	Ti	Ti121	0.59725	0.63877	0.23180	1.000	0.000	1a	1
122	Ti	Ti122	0.49055	0.13681	0.23356	1.000	0.000	1a	1
123	Ti	Ti123	0.50041	0.40532	0.21483	1.000	0.000	1a	1
124	Ti	Ti124	0.58905	-0.09340	0.21275	1.000	0.000	1a	1
125	O	O125	0.59848	0.44264	0.21690	1.000	0.000	1a	1
126	O	O126	0.48805	-0.05569	0.21821	1.000	0.000	1a	1
127	O	O127	0.48769	0.23007	0.18280	1.000	0.000	1a	1
128	O	O128	0.59713	0.73154	0.18004	1.000	0.000	1a	1
129	O	O129	0.59195	0.08525	0.23514	1.000	0.000	1a	1
130	O	O130	0.49915	0.58744	0.23744	1.000	0.000	1a	1
131	O	O131	0.50029	0.29191	0.26339	1.000	0.000	1a	1

132 O	O132	0.59150	-0.20279	0.26106	1.000	0.000	1a	1
133 Ti	Ti133	0.79336	0.63681	0.23234	1.000	0.000	1a	1
134 Ti	Ti134	0.69367	0.12768	0.23113	1.000	0.000	1a	1
135 Ti	Ti135	0.69558	0.39891	0.21454	1.000	0.000	1a	1
136 Ti	Ti136	0.79679	-0.09596	0.21470	1.000	0.000	1a	1
137 O	O137	0.79245	0.44330	0.21727	1.000	0.000	1a	1
138 O	O138	0.69349	-0.06035	0.21372	1.000	0.000	1a	1
139 O	O139	0.69485	0.22466	0.18171	1.000	0.000	1a	1
140 O	O140	0.79631	0.73256	0.18099	1.000	0.000	1a	1
141 O	O141	0.79550	0.08329	0.23633	1.000	0.000	1a	1
142 O	O142	0.69515	0.58965	0.23922	1.000	0.000	1a	1
143 O	O143	0.69485	0.28355	0.26225	1.000	0.000	1a	1
144 O	O144	0.79398	-0.20474	0.26258	1.000	0.000	1a	1
145 Ti	Ti145	0.99146	0.63352	0.23500	1.000	0.000	1a	1
146 Ti	Ti146	0.89651	0.13236	0.23388	1.000	0.000	1a	1
147 Ti	Ti147	0.89120	0.40191	0.21624	1.000	0.000	1a	1
148 Ti	Ti148	0.99764	-0.09743	0.21679	1.000	0.000	1a	1
149 O	O149	0.98931	0.44043	0.21931	1.000	0.000	1a	1
150 O	O150	0.89856	-0.06070	0.21882	1.000	0.000	1a	1
151 O	O151	0.90070	0.22742	0.18369	1.000	0.000	1a	1
152 O	O152	0.99021	0.72730	0.18437	1.000	0.000	1a	1
153 O	O153	0.99668	0.08591	0.24085	1.000	0.000	1a	1
154 O	O154	0.89125	0.58640	0.24088	1.000	0.000	1a	1
155 O	O155	0.88915	0.28732	0.26438	1.000	0.000	1a	1
156 O	O156	0.99772	-0.21076	0.26518	1.000	0.000	1a	1
157 S	S157	0.36813	-0.24520	0.32206	1.000	0.000	1a	1
158 O	O158	0.42214	-0.18621	0.35090	1.000	0.000	1a	1
159 O	O159	0.39205	-0.38980	0.30584	1.000	0.000	1a	1

==

SOF_2/TiO_2-ZnO

==

Title SOF_2/TiO_2-ZnO

Lattice type P
Space group name P 1
Space group number 1
Setting number 1

Lattice parameters

a	b	c	alpha	beta	gamma
19.18780	10.31040	29.67230	90.0000	90.0000	90.0000

Unit-cell volume = 5870.186532 Å^3

Structure parameters

		x	y	z	Occ.	U	Site	Sym.
1 Zn	Zn1	0.02654	0.02883	0.01820	1.000	0.000	1a	1
2 Zn	Zn2	0.10706	1.01078	0.11111	1.000	0.000	1a	1
3 O	O3	0.02431	0.19474	-0.00767	1.000	0.000	1a	1
4 O	O4	0.10896	0.19031	0.08796	1.000	0.000	1a	1
5 Zn	Zn5	0.10877	0.26055	0.01926	1.000	0.000	1a	1
6 Zn	Zn6	0.03056	0.26787	0.12198	1.000	0.000	1a	1
7 O	O7	0.10868	0.45111	0.02726	1.000	0.000	1a	1
8 O	O8	0.03534	0.45231	0.13256	1.000	0.000	1a	1
9 Zn	Zn9	0.19231	0.02926	0.01827	1.000	0.000	1a	1
10 Zn	Zn10	0.28079	1.01132	0.10966	1.000	0.000	1a	1
11 O	O11	0.19247	0.19397	-0.00871	1.000	0.000	1a	1

12 O	O12	0.27591	0.19174	0.08702	1.000	0.000	1a	1
13 Zn	Zn13	0.27596	0.26172	0.01919	1.000	0.000	1a	1
14 Zn	Zn14	0.19256	0.26779	0.11360	1.000	0.000	1a	1
15 O	O15	0.27692	0.45138	0.02981	1.000	0.000	1a	1
16 O	O16	0.19131	0.44812	0.12372	1.000	0.000	1a	1
17 Zn	Zn17	0.35712	0.02979	0.01352	1.000	0.000	1a	1
18 Zn	Zn18	0.44700	1.01398	0.10361	1.000	0.000	1a	1
19 O	O19	0.36031	0.19948	-0.00925	1.000	0.000	1a	1
20 O	O20	0.43855	0.20243	0.09112	1.000	0.000	1a	1
21 Zn	Zn21	0.44046	0.26298	0.02391	1.000	0.000	1a	1
22 Zn	Zn22	0.35507	0.26827	0.12033	1.000	0.000	1a	1
23 O	O23	0.44092	0.44948	0.04065	1.000	0.000	1a	1
24 O	O24	0.34797	0.45188	0.13705	1.000	0.000	1a	1
25 Zn	Zn25	0.52311	0.03203	0.00756	1.000	0.000	1a	1
26 Zn	Zn26	0.60943	1.01269	0.09500	1.000	0.000	1a	1
27 O	O27	0.52438	0.20884	-0.00765	1.000	0.000	1a	1
28 O	O28	0.60799	0.20493	0.08968	1.000	0.000	1a	1
29 Zn	Zn29	0.60877	0.26409	0.02204	1.000	0.000	1a	1
30 Zn	Zn30	0.52458	0.26702	0.11924	1.000	0.000	1a	1
31 O	O31	0.60928	0.44684	0.04321	1.000	0.000	1a	1
32 O	O32	0.51921	0.45119	0.14238	1.000	0.000	1a	1
33 Zn	Zn33	0.69399	0.03131	0.00590	1.000	0.000	1a	1
34 Zn	Zn34	0.77422	1.01408	0.09849	1.000	0.000	1a	1
35 O	O35	0.69391	0.20962	-0.00732	1.000	0.000	1a	1
36 O	O36	0.78029	0.20458	0.09114	1.000	0.000	1a	1
37 Zn	Zn37	0.77816	0.26369	0.02328	1.000	0.000	1a	1
38 Zn	Zn38	0.69325	0.26725	0.11712	1.000	0.000	1a	1
39 O	O39	0.77721	0.44751	0.04286	1.000	0.000	1a	1
40 O	O40	0.69483	0.45122	0.14238	1.000	0.000	1a	1
41 Zn	Zn41	0.86315	0.03227	0.01091	1.000	0.000	1a	1
42 Zn	Zn42	0.93607	1.01304	0.10948	1.000	0.000	1a	1
43 O	O43	0.86183	0.20658	-0.00687	1.000	0.000	1a	1
44 O	O44	0.94708	0.19787	0.09225	1.000	0.000	1a	1
45 Zn	Zn45	0.94503	0.26279	0.02525	1.000	0.000	1a	1
46 Zn	Zn46	0.86288	0.26888	0.12131	1.000	0.000	1a	1
47 O	O47	0.94394	0.45127	0.03807	1.000	0.000	1a	1
48 O	O48	0.86894	0.45251	0.14179	1.000	0.000	1a	1
49 Zn	Zn49	0.02250	0.53318	0.00792	1.000	0.000	1a	1
50 Zn	Zn50	0.10991	0.51353	0.09442	1.000	0.000	1a	1
51 O	O51	0.02375	0.70970	-0.00738	1.000	0.000	1a	1
52 O	O52	0.10772	0.70553	0.08971	1.000	0.000	1a	1
53 Zn	Zn53	0.10857	0.76465	0.02191	1.000	0.000	1a	1
54 Zn	Zn54	0.02508	0.76848	0.11995	1.000	0.000	1a	1
55 O	O55	0.10923	0.94712	0.04305	1.000	0.000	1a	1
56 O	O56	0.01968	0.95226	0.14284	1.000	0.000	1a	1
57 Zn	Zn57	0.19357	0.53180	0.00515	1.000	0.000	1a	1
58 Zn	Zn58	0.27423	0.51500	0.09707	1.000	0.000	1a	1
59 O	O59	0.19331	0.71012	-0.00787	1.000	0.000	1a	1
60 O	O60	0.28008	0.70546	0.09010	1.000	0.000	1a	1
61 Zn	Zn61	0.27817	0.76406	0.02214	1.000	0.000	1a	1
62 Zn	Zn62	0.19349	0.76929	0.11636	1.000	0.000	1a	1
63 O	O63	0.27684	0.94796	0.04153	1.000	0.000	1a	1
64 O	O64	0.19470	0.95277	0.14200	1.000	0.000	1a	1
65 Zn	Zn65	0.36328	0.53208	0.00981	1.000	0.000	1a	1
66 Zn	Zn66	0.43602	0.51247	0.10863	1.000	0.000	1a	1
67 O	O67	0.36190	0.70646	-0.00780	1.000	0.000	1a	1
68 O	O68	0.44699	0.69719	0.09159	1.000	0.000	1a	1
69 Zn	Zn69	0.44497	0.76250	0.02464	1.000	0.000	1a	1
70 Zn	Zn70	0.36287	0.76878	0.12051	1.000	0.000	1a	1
71 O	O71	0.44275	0.95148	0.03611	1.000	0.000	1a	1
72 O	O72	0.36800	0.95234	0.14080	1.000	0.000	1a	1
73 Zn	Zn73	0.52672	0.52834	0.01793	1.000	0.000	1a	1

74	Zn	Zn74	0.60707	0.50927	0.11121	1.000	0.000	1a	1
75	O	O75	0.52523	0.69491	-0.00739	1.000	0.000	1a	1
76	O	O76	0.60923	0.68993	0.08945	1.000	0.000	1a	1
77	Zn	Zn77	0.60910	0.75996	0.02062	1.000	0.000	1a	1
78	Zn	Zn78	0.52990	0.76809	0.12249	1.000	0.000	1a	1
79	O	O79	0.60903	0.95068	0.02759	1.000	0.000	1a	1
80	O	O80	0.53206	0.95278	0.13023	1.000	0.000	1a	1
81	Zn	Zn81	0.69294	0.52851	0.01902	1.000	0.000	1a	1
82	Zn	Zn82	0.78124	0.51122	0.11079	1.000	0.000	1a	1
83	O	O83	0.69297	0.69383	-0.00736	1.000	0.000	1a	1
84	O	O84	0.77613	0.69209	0.08878	1.000	0.000	1a	1
85	Zn	Zn85	0.77623	0.76138	0.02081	1.000	0.000	1a	1
86	Zn	Zn86	0.69254	0.76825	0.11519	1.000	0.000	1a	1
87	O	O87	0.77710	0.95113	0.03111	1.000	0.000	1a	1
88	O	O88	0.69076	0.94879	0.12503	1.000	0.000	1a	1
89	Zn	Zn89	0.85799	0.52943	0.01558	1.000	0.000	1a	1
90	Zn	Zn90	0.94927	0.51380	0.10580	1.000	0.000	1a	1
91	O	O91	0.86041	0.69851	-0.00769	1.000	0.000	1a	1
92	O	O92	0.93913	0.70139	0.09217	1.000	0.000	1a	1
93	Zn	Zn93	0.94052	0.76348	0.02509	1.000	0.000	1a	1
94	Zn	Zn94	0.85592	0.76764	0.12191	1.000	0.000	1a	1
95	O	O95	0.94108	0.94996	0.04160	1.000	0.000	1a	1
96	O	O96	0.84857	0.95169	0.13816	1.000	0.000	1a	1
97	Ti	Ti97	0.19946	0.63289	0.23124	1.000	0.000	1a	1
98	Ti	Ti98	0.09978	0.13852	0.23233	1.000	0.000	1a	1
99	Ti	Ti99	0.09752	0.40576	0.21740	1.000	0.000	1a	1
100	Ti	Ti100	0.19915	-0.09598	0.21427	1.000	0.000	1a	1
101	O	O101	0.19975	0.44166	0.21517	1.000	0.000	1a	1
102	O	O102	0.10097	-0.05361	0.21767	1.000	0.000	1a	1
103	O	O103	0.09595	0.23943	0.18209	1.000	0.000	1a	1
104	O	O104	0.19917	0.72875	0.18163	1.000	0.000	1a	1
105	O	O105	0.19891	0.09271	0.23816	1.000	0.000	1a	1
106	O	O106	0.09827	0.58715	0.23722	1.000	0.000	1a	1
107	O	O107	0.10020	0.29493	0.26438	1.000	0.000	1a	1
108	O	O108	0.19913	-0.21216	0.26222	1.000	0.000	1a	1
109	Ti	Ti109	0.40133	0.63749	0.23292	1.000	0.000	1a	1
110	Ti	Ti110	0.29757	0.13978	0.23113	1.000	0.000	1a	1
111	Ti	Ti111	0.30284	0.40755	0.21313	1.000	0.000	1a	1
112	Ti	Ti112	0.39647	-0.09330	0.21550	1.000	0.000	1a	1
113	O	O113	0.40351	0.44332	0.21797	1.000	0.000	1a	1
114	O	O114	0.29658	-0.05497	0.21770	1.000	0.000	1a	1
115	O	O115	0.29749	0.23480	0.17992	1.000	0.000	1a	1
116	O	O116	0.40643	0.73338	0.18262	1.000	0.000	1a	1
117	O	O117	0.39688	0.08975	0.23799	1.000	0.000	1a	1
118	O	O118	0.30114	0.58787	0.23531	1.000	0.000	1a	1
119	O	O119	0.30158	0.29685	0.26127	1.000	0.000	1a	1
120	O	O120	0.39411	-0.20781	0.26352	1.000	0.000	1a	1
121	Ti	Ti121	0.59862	0.63600	0.23280	1.000	0.000	1a	1
122	Ti	Ti122	0.49794	0.13431	0.23350	1.000	0.000	1a	1
123	Ti	Ti123	0.50147	0.40368	0.21548	1.000	0.000	1a	1
124	Ti	Ti124	0.59927	-0.09604	0.22171	1.000	0.000	1a	1
125	O	O125	0.60047	0.44584	0.21756	1.000	0.000	1a	1
126	O	O126	0.49685	-0.05828	0.21998	1.000	0.000	1a	1
127	O	O127	0.49761	0.22864	0.18320	1.000	0.000	1a	1
128	O	O128	0.59448	0.74335	0.18408	1.000	0.000	1a	1
129	O	O129	0.59876	0.08645	0.24043	1.000	0.000	1a	1
130	O	O130	0.50096	0.58850	0.24083	1.000	0.000	1a	1
131	O	O131	0.50165	0.29045	0.26384	1.000	0.000	1a	1
132	O	O132	0.60130	-0.21047	0.26739	1.000	0.000	1a	1
133	Ti	Ti133	0.79719	0.63983	0.23260	1.000	0.000	1a	1
134	Ti	Ti134	0.69947	0.13394	0.23273	1.000	0.000	1a	1
135	Ti	Ti135	0.69901	0.40428	0.21408	1.000	0.000	1a	1

136	Ti	Ti136	0.80360	-0.09237	0.21507	1.000	0.000	1a	1
137	O	O137	0.79613	0.44544	0.21805	1.000	0.000	1a	1
138	O	O138	0.69990	-0.05858	0.21733	1.000	0.000	1a	1
139	O	O139	0.69801	0.22727	0.18252	1.000	0.000	1a	1
140	O	O140	0.79827	0.73588	0.18160	1.000	0.000	1a	1
141	O	O141	0.80106	0.08867	0.23653	1.000	0.000	1a	1
142	O	O142	0.69834	0.59223	0.23761	1.000	0.000	1a	1
143	O	O143	0.69901	0.29034	0.26285	1.000	0.000	1a	1
144	O	O144	0.80129	-0.20353	0.26303	1.000	0.000	1a	1
145	Ti	Ti145	0.99715	0.63537	0.23349	1.000	0.000	1a	1
146	Ti	Ti146	0.90169	0.13819	0.23385	1.000	0.000	1a	1
147	Ti	Ti147	0.89579	0.40700	0.21611	1.000	0.000	1a	1
148	Ti	Ti148	1.00227	-0.09489	0.21620	1.000	0.000	1a	1
149	O	O149	0.99552	0.44228	0.21949	1.000	0.000	1a	1
150	O	O150	0.90366	-0.05632	0.21993	1.000	0.000	1a	1
151	O	O151	0.90535	0.23305	0.18338	1.000	0.000	1a	1
152	O	O152	0.99421	0.73101	0.18347	1.000	0.000	1a	1
153	O	O153	1.00181	0.09010	0.24055	1.000	0.000	1a	1
154	O	O154	0.89631	0.58960	0.23926	1.000	0.000	1a	1
155	O	O155	0.89427	0.29277	0.26433	1.000	0.000	1a	1
156	O	O156	1.00358	-0.20994	0.26425	1.000	0.000	1a	1
157	S	S157	0.61762	0.53544	0.32991	1.000	0.000	1a	1
158	F	F158	0.55699	0.60377	0.36163	1.000	0.000	1a	1
159	F	F159	0.59545	0.38717	0.34505	1.000	0.000	1a	1
160	O	O160	0.68514	0.56483	0.34989	1.000	0.000	1a	1

==

SO_2F_2/TiO_2-ZnO

==

Title SO_2F_2/TiO_2-ZnO

Lattice type P
Space group name P 1
Space group number 1
Setting number 1

Lattice parameters

a	b	c	alpha	beta	gamma
19.18780	10.31040	29.67230	90.0000	90.0000	90.0000

Unit-cell volume = 5870.186532 Å^3

Structure parameters

			x	y	z	Occ.	U	Site	Sym.
1	Zn	Zn1	0.03159	0.02593	0.01707	1.000	0.000	1a	1
2	Zn	Zn2	0.10916	1.00806	0.11173	1.000	0.000	1a	1
3	O	O3	0.02970	0.19411	-0.00698	1.000	0.000	1a	1
4	O	O4	0.11376	0.18892	0.08969	1.000	0.000	1a	1
5	Zn	Zn5	0.11360	0.25789	0.02168	1.000	0.000	1a	1
6	Zn	Zn6	0.03360	0.26432	0.12267	1.000	0.000	1a	1
7	O	O7	0.11288	0.44806	0.03096	1.000	0.000	1a	1
8	O	O8	0.04037	0.44876	0.13703	1.000	0.000	1a	1
9	Zn	Zn9	0.19692	0.02552	0.01976	1.000	0.000	1a	1
10	Zn	Zn10	0.28313	1.00682	0.11191	1.000	0.000	1a	1
11	O	O11	0.19692	0.19081	-0.00660	1.000	0.000	1a	1
12	O	O12	0.28065	0.18764	0.09016	1.000	0.000	1a	1
13	Zn	Zn13	0.28070	0.25703	0.02146	1.000	0.000	1a	1
14	Zn	Zn14	0.19732	0.26575	0.11606	1.000	0.000	1a	1

15 O	O15	0.28096	0.44788	0.02825	1.000	0.000	1a	1
16 O	O16	0.19871	0.44650	0.12551	1.000	0.000	1a	1
17 Zn	Zn17	0.36304	0.02558	0.01823	1.000	0.000	1a	1
18 Zn	Zn18	0.45427	1.00926	0.10815	1.000	0.000	1a	1
19 O	O19	0.36412	0.19195	-0.00730	1.000	0.000	1a	1
20 O	O20	0.44285	0.19401	0.09089	1.000	0.000	1a	1
21 Zn	Zn21	0.44468	0.26022	0.02409	1.000	0.000	1a	1
22 Zn	Zn22	0.36069	0.26411	0.12303	1.000	0.000	1a	1
23 O	O23	0.44706	0.44900	0.03626	1.000	0.000	1a	1
24 O	O24	0.35791	0.44898	0.13088	1.000	0.000	1a	1
25 Zn	Zn25	0.52611	0.02976	0.00854	1.000	0.000	1a	1
26 Zn	Zn26	0.61556	1.01151	0.09502	1.000	0.000	1a	1
27 O	O27	0.52753	0.20443	-0.00874	1.000	0.000	1a	1
28 O	O28	0.61022	0.20205	0.08890	1.000	0.000	1a	1
29 Zn	Zn29	0.61190	0.26095	0.02066	1.000	0.000	1a	1
30 Zn	Zn30	0.52745	0.26480	0.11922	1.000	0.000	1a	1
31 O	O31	0.61320	0.44438	0.04038	1.000	0.000	1a	1
32 O	O32	0.52249	0.44843	0.13983	1.000	0.000	1a	1
33 Zn	Zn33	0.69685	0.02841	0.00416	1.000	0.000	1a	1
34 Zn	Zn34	0.77988	1.01077	0.09367	1.000	0.000	1a	1
35 O	O35	0.69707	0.20677	-0.00884	1.000	0.000	1a	1
36 O	O36	0.78294	0.20242	0.08893	1.000	0.000	1a	1
37 Zn	Zn37	0.78182	0.26153	0.02100	1.000	0.000	1a	1
38 Zn	Zn38	0.69678	0.26717	0.11496	1.000	0.000	1a	1
39 O	O39	0.78060	0.44401	0.04182	1.000	0.000	1a	1
40 O	O40	0.69574	0.45049	0.14055	1.000	0.000	1a	1
41 Zn	Zn41	0.86812	0.03039	0.00783	1.000	0.000	1a	1
42 Zn	Zn42	0.93952	1.01030	0.10713	1.000	0.000	1a	1
43 O	O43	0.86688	0.20639	-0.00797	1.000	0.000	1a	1
44 O	O44	0.95064	0.19692	0.09230	1.000	0.000	1a	1
45 Zn	Zn45	0.94946	0.26027	0.02538	1.000	0.000	1a	1
46 Zn	Zn46	0.86487	0.26570	0.12003	1.000	0.000	1a	1
47 O	O47	0.94843	0.44734	0.04083	1.000	0.000	1a	1
48 O	O48	0.87028	0.44953	0.14214	1.000	0.000	1a	1
49 Zn	Zn49	0.02698	0.52934	0.01070	1.000	0.000	1a	1
50 Zn	Zn50	0.11543	0.51068	0.09835	1.000	0.000	1a	1
51 O	O51	0.02844	0.70420	-0.00658	1.000	0.000	1a	1
52 O	O52	0.11030	0.70179	0.09150	1.000	0.000	1a	1
53 Zn	Zn53	0.11210	0.76075	0.02374	1.000	0.000	1a	1
54 Zn	Zn54	0.02743	0.76579	0.12119	1.000	0.000	1a	1
55 O	O55	0.11284	0.94427	0.04377	1.000	0.000	1a	1
56 O	O56	0.02085	0.94963	0.14234	1.000	0.000	1a	1
57 Zn	Zn57	0.19619	0.52846	0.00622	1.000	0.000	1a	1
58 Zn	Zn58	0.28041	0.50977	0.09573	1.000	0.000	1a	1
59 O	O59	0.19613	0.70674	-0.00712	1.000	0.000	1a	1
60 O	O60	0.28196	0.70190	0.08997	1.000	0.000	1a	1
61 Zn	Zn61	0.28100	0.76141	0.02250	1.000	0.000	1a	1
62 Zn	Zn62	0.19701	0.76429	0.11803	1.000	0.000	1a	1
63 O	O63	0.28079	0.94409	0.04378	1.000	0.000	1a	1
64 O	O64	0.19579	0.94851	0.14324	1.000	0.000	1a	1
65 Zn	Zn65	0.36649	0.52975	0.00788	1.000	0.000	1a	1
66 Zn	Zn66	0.44229	0.51124	0.10362	1.000	0.000	1a	1
67 O	O67	0.36533	0.70642	-0.00739	1.000	0.000	1a	1
68 O	O68	0.45124	0.69980	0.09139	1.000	0.000	1a	1
69 Zn	Zn69	0.44949	0.76012	0.02399	1.000	0.000	1a	1
70 Zn	Zn70	0.36516	0.76553	0.11942	1.000	0.000	1a	1
71 O	O71	0.44946	0.94682	0.04035	1.000	0.000	1a	1
72 O	O72	0.37166	0.94969	0.14234	1.000	0.000	1a	1
73 Zn	Zn73	0.53258	0.52707	0.01317	1.000	0.000	1a	1
74 Zn	Zn74	0.60974	0.50852	0.10846	1.000	0.000	1a	1
75 O	O75	0.52866	0.69612	-0.01011	1.000	0.000	1a	1
76 O	O76	0.61340	0.68813	0.08496	1.000	0.000	1a	1

77	Zn	Zn77	0.61364	0.75879	0.01734	1.000	0.000	1a	1
78	Zn	Zn78	0.53553	0.76343	0.12000	1.000	0.000	1a	1
79	O	O79	0.61267	0.94852	0.02786	1.000	0.000	1a	1
80	O	O80	0.54320	0.94826	0.13631	1.000	0.000	1a	1
81	Zn	Zn81	0.69760	0.52663	0.01718	1.000	0.000	1a	1
82	Zn	Zn82	0.78346	0.50832	0.10999	1.000	0.000	1a	1
83	O	O83	0.69743	0.69098	-0.01017	1.000	0.000	1a	1
84	O	O84	0.78113	0.68728	0.08617	1.000	0.000	1a	1
85	Zn	Zn85	0.78122	0.75769	0.01779	1.000	0.000	1a	1
86	Zn	Zn86	0.69699	0.76299	0.11182	1.000	0.000	1a	1
87	O	O87	0.78162	0.94805	0.02661	1.000	0.000	1a	1
88	O	O88	0.69804	0.94351	0.12144	1.000	0.000	1a	1
89	Zn	Zn89	0.86324	0.52598	0.01701	1.000	0.000	1a	1
90	Zn	Zn90	0.95371	0.51044	0.10868	1.000	0.000	1a	1
91	O	O91	0.86616	0.69222	-0.00865	1.000	0.000	1a	1
92	O	O92	0.94281	0.69612	0.09213	1.000	0.000	1a	1
93	Zn	Zn93	0.94481	0.75979	0.02512	1.000	0.000	1a	1
94	Zn	Zn94	0.85886	0.76430	0.12117	1.000	0.000	1a	1
95	O	O95	0.94526	0.94773	0.03926	1.000	0.000	1a	1
96	O	O96	0.85228	0.94886	0.13397	1.000	0.000	1a	1
97	Ti	Ti97	0.19206	0.63011	0.23349	1.000	0.000	1a	1
98	Ti	Ti98	0.09439	0.13619	0.23320	1.000	0.000	1a	1
99	Ti	Ti99	0.08871	0.40399	0.21690	1.000	0.000	1a	1
100	Ti	Ti100	0.19305	-0.09925	0.21547	1.000	0.000	1a	1
101	O	O101	0.19184	0.43790	0.21824	1.000	0.000	1a	1
102	O	O102	0.09571	-0.05730	0.21815	1.000	0.000	1a	1
103	O	O103	0.09293	0.23426	0.18257	1.000	0.000	1a	1
104	O	O104	0.19448	0.72448	0.18348	1.000	0.000	1a	1
105	O	O105	0.19312	0.08929	0.23971	1.000	0.000	1a	1
106	O	O106	0.09060	0.58522	0.23768	1.000	0.000	1a	1
107	O	O107	0.09046	0.29247	0.26441	1.000	0.000	1a	1
108	O	O108	0.19196	-0.21381	0.26386	1.000	0.000	1a	1
109	Ti	Ti109	0.39303	0.63219	0.23370	1.000	0.000	1a	1
110	Ti	Ti110	0.29253	0.13421	0.23386	1.000	0.000	1a	1
111	Ti	Ti111	0.29366	0.40159	0.22240	1.000	0.000	1a	1
112	Ti	Ti112	0.38997	-0.09809	0.21581	1.000	0.000	1a	1
113	O	O113	0.39490	0.43992	0.22010	1.000	0.000	1a	1
114	O	O114	0.29108	-0.05625	0.21854	1.000	0.000	1a	1
115	O	O115	0.29623	0.24017	0.18489	1.000	0.000	1a	1
116	O	O116	0.38825	0.72721	0.18367	1.000	0.000	1a	1
117	O	O117	0.39072	0.08694	0.24114	1.000	0.000	1a	1
118	O	O118	0.29275	0.58342	0.24173	1.000	0.000	1a	1
119	O	O119	0.29184	0.28742	0.26816	1.000	0.000	1a	1
120	O	O120	0.39321	-0.21180	0.26428	1.000	0.000	1a	1
121	Ti	Ti121	0.59463	0.63960	0.22975	1.000	0.000	1a	1
122	Ti	Ti122	0.48956	0.13735	0.23177	1.000	0.000	1a	1
123	Ti	Ti123	0.49593	0.40646	0.21270	1.000	0.000	1a	1
124	Ti	Ti124	0.58820	-0.09219	0.20978	1.000	0.000	1a	1
125	O	O125	0.59520	0.44359	0.21593	1.000	0.000	1a	1
126	O	O126	0.48735	-0.05768	0.21604	1.000	0.000	1a	1
127	O	O127	0.48248	0.23155	0.18111	1.000	0.000	1a	1
128	O	O128	0.59626	0.73162	0.17772	1.000	0.000	1a	1
129	O	O129	0.59033	0.08625	0.23255	1.000	0.000	1a	1
130	O	O130	0.49478	0.59108	0.23273	1.000	0.000	1a	1
131	O	O131	0.49942	0.29207	0.26148	1.000	0.000	1a	1
132	O	O132	0.58916	-0.20152	0.25855	1.000	0.000	1a	1
133	Ti	Ti133	0.79138	0.63739	0.23091	1.000	0.000	1a	1
134	Ti	Ti134	0.69247	0.12976	0.22943	1.000	0.000	1a	1
135	Ti	Ti135	0.69253	0.40135	0.21347	1.000	0.000	1a	1
136	Ti	Ti136	0.79513	-0.09511	0.21368	1.000	0.000	1a	1
137	O	O137	0.79050	0.44344	0.21712	1.000	0.000	1a	1
138	O	O138	0.69242	-0.05918	0.21239	1.000	0.000	1a	1

139 O	O139	0.69361	0.22746	0.18027	1.000	0.000	1a	1
140 O	O140	0.79506	0.73400	0.17980	1.000	0.000	1a	1
141 O	O141	0.79392	0.08480	0.23514	1.000	0.000	1a	1
142 O	O142	0.69290	0.59087	0.23694	1.000	0.000	1a	1
143 O	O143	0.69180	0.28442	0.26100	1.000	0.000	1a	1
144 O	O144	0.79172	-0.20461	0.26147	1.000	0.000	1a	1
145 Ti	Ti145	0.98987	0.63492	0.23388	1.000	0.000	1a	1
146 Ti	Ti146	0.89502	0.13350	0.23312	1.000	0.000	1a	1
147 Ti	Ti147	0.88920	0.40323	0.21598	1.000	0.000	1a	1
148 Ti	Ti148	0.99604	-0.09615	0.21610	1.000	0.000	1a	1
149 O	O149	0.98813	0.44051	0.22027	1.000	0.000	1a	1
150 O	O150	0.89699	-0.05972	0.21858	1.000	0.000	1a	1
151 O	O151	0.89964	0.22950	0.18316	1.000	0.000	1a	1
152 O	O152	0.98764	0.72933	0.18343	1.000	0.000	1a	1
153 O	O153	0.99555	0.08658	0.24004	1.000	0.000	1a	1
154 O	O154	0.88952	0.58775	0.23969	1.000	0.000	1a	1
155 O	O155	0.88707	0.28757	0.26397	1.000	0.000	1a	1
156 O	O156	0.99672	-0.21044	0.26441	1.000	0.000	1a	1
157 S	S157	0.50907	0.57940	0.34059	1.000	0.000	1a	1
158 O	O158	0.44067	0.53652	0.32684	1.000	0.000	1a	1
159 O	O159	0.57422	0.55377	0.31763	1.000	0.000	1a	1
160 F	F160	0.51976	0.53710	0.39193	1.000	0.000	1a	1
161 F	F161	0.50516	0.73128	0.35064	1.000	0.000	1a	1

==
